# The asymmetrical ESR1 signaling in muscle progenitor cells determines the progression of adolescent idiopathic scoliosis

**DOI:** 10.1038/s41421-023-00531-5

**Published:** 2023-04-25

**Authors:** Xiexiang Shao, Xin Fu, Jingfan Yang, Wenyuan Sui, Sheng Li, Wenjun Yang, Xingzuan Lin, Yuanyuan Zhang, Minzhi Jia, Huan Liu, Wei Liu, Lili Han, Yang Yu, Yaolong Deng, Tianyuan Zhang, Junlin Yang, Ping Hu

**Affiliations:** 1 Xinhua Hospital Affiliated to Shanghai Jiao Tong University School of Medicine, Shanghai, China; 2 State Key Laboratory of Cell Biology, Shanghai Institute of Biochemistry and Cell Biology, Center for Excellence in Molecular Cell Science, Chinese Academy of Sciences, Shanghai, China; 3 Centre Testing International Medical Laboratory (CTI-Medlab), Shanghai, China; 4 Guangzhou Laboratory, Guangzhou International Bio Island, Guangzhou, Guangdong, China; 5 Institute for Stem Cell and Regeneration, Chinese Academy of Sciences, Beijing, China; 6 Key Laboratory of Biological Targeting Diagnosis, Therapy and Rehabilitation of Guangdong Higher Education Institutes, The Fifth Affiliated Hospital of Guangzhou Medical University, Guangzhou, Guangdong, China

**Keywords:** Muscle stem cells, Stem-cell differentiation

## Abstract

Adolescent Idiopathic Scoliosis (AIS) is a common pediatric skeletal disease highly occurred in females. The pathogenesis of AIS has not been fully elucidated. Here, we reveal that ESR1 (Estrogen Receptor 1) expression declines in muscle stem/progenitor cells at the concave side of AIS patients. Furthermore, ESR1 is required for muscle stem/progenitor cell differentiation and disrupted ESR1 signaling leads to differentiation defects. The imbalance of ESR1 signaling in the para-spinal muscles induces scoliosis in mice, while reactivation of ESR1 signaling at the concave side by an FDA approved drug Raloxifene alleviates the curve progression. This work reveals that the asymmetric inactivation of ESR1 signaling is one of the causes of AIS. Reactivation of ESR1 signaling in para-spinal muscle by Raloxifene at the concave side could be a new strategy to treat AIS.

Adolescent idiopathic scoliosis (AIS) is a complex three-dimensional spinal deformity with spinal curvature >10°^1,2^. As a common pediatric skeletal disease, it develops in early adolescence and affects ~1–4% of adolescents worldwide. Disproportionately high percentage of females suffer AIS (female/male ratio: 3:1), and female patients also tend to have higher radius of curvature^3^. Patients with severe curve could even suffer cardiorespiratory dysfunction and other health problems^4,5^. Although genetic^6,7^, environmental^8^, neuromuscular^9,10^, and hormone factors^11^ have all been linked to AIS, the mechanism of the initiation and progression of AIS remains to be elucidated.

Many studies focus on the defects of bone development to identify the causes of AIS^11–13^. The functions of the non-bone cells in AIS have not been fully understood. Para-vertebral muscles are among the most important subsystems for spinal stability and key for the balanced spinal loading and alignment^14–17^. The imbalance of muscle force of the para-spinal muscles is attributed to not only the initiation of the spine instability but also the further progress of abnormal curvature in AIS. Based on the Hueter-Volkmann’s law, which describes the general mechanism of the spinal deformation and scoliosis, decreased mechanical loading on the spinal bone structure leads to increased bone and soft tissue growth; while excessive loading on the bone structure leads to growth retardation^15^. The strong para-spinal muscles reduce the mechanical loading at the convex side, which causes further bone and soft tissue growth to increase the curvature. Likewise, the weak para-spinal muscles render more mechanical loading on the skeletal structure at the concave side which impedes the growth of bone and soft tissue. Imbalanced bilateral para-spinal muscles could first apply asymmetric loading to spine and lead to spinal instability and curvature^18^. The asymmetrical growth of the bone structure further aggravates the abnormal spinal curvature and AIS. Consistent with the theory, the para-spinal muscle asymmetry detected by magnetic resonance imaging^16,19^, biomechanical tests^20^, and muscle histology^14^ has been reported to be associated with AIS. And the asymmetrical functions of the para-vertebral muscles have been reported to closely correlate with the curve severity of AIS^20^.

Myogenesis mediated by muscle stem cells is critical for skeletal muscle growth and regeneration^21–26^. Genome-wide association analysis has identified *PAX3* and *MYOD1*, which encode key transcription factors regulating muscle stem cell functions and myogenesis^27^, as potential susceptible loci for AIS^7,28,29^. The asymmetric expression of *PAX3* and *MYOD1* in AIS patients has also been reported. These studies indicate that muscle stem cells and myogenesis may be involved in AIS. However, the direct evidence linking the defects of myogenesis and AIS is missing.

Here, we found that ESR1 is required for human muscle stem/progenitor cell differentiation by activating AKT-CREB signaling. The expression level of ESR1 decreased in muscle stem/progenitor cells at the concave side in AIS patients, which resulted in differentiation defects and aggravating scoliosis. We built a scoliosis mouse model by inhibiting ESR1 activity at one side of the para-spinal muscle, and reactivation of ESR1 by an FDA approved drug Raloxifene at the concave side greatly alleviates the progress of scoliosis. These findings establish the link between myogenesis defects and AIS, and provide a new potential treatment strategy for AIS.

## Results

### ESR1 expression decreases in muscle stem/progenitor cells at the concave side of AIS patients

**Fig. 1:**
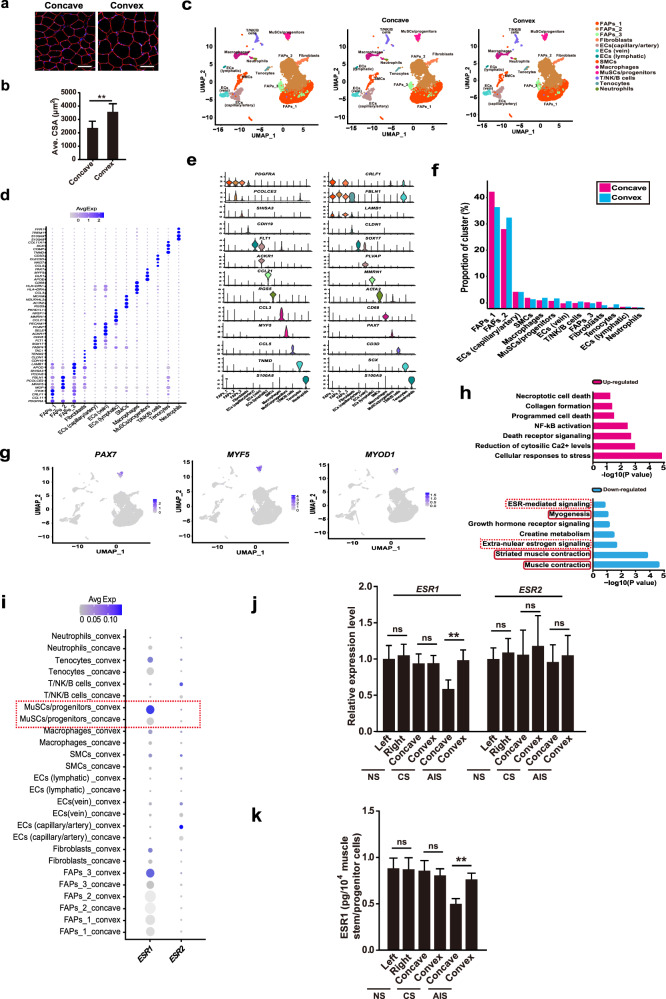
ESR1 expression decreases in muscle stem/progenitor cells at the concave side of AIS patients. **a** Representative immunofluorescent staining of bilateral para-spinal muscle sections from an AIS patient. Cryosections were obtained from the convex and concave sides of AIS patients. The cryosections were stained with anti-Laminin antibody. Red indicated laminin; Blue indicated DAPI staining of nuclei. The merged images were shown. Scale bars: 100 μm. **b** Statistical analysis of the cross-sectional area (CSA) of myofibers. At least 350 fibers were analyzed for each sample. Error bars indicated standard deviation (*n* = 6). **P < 0.01. **c** Muscle tissues harvested from the concave and convex side of AIS patients were subjected for scRNA-seq. Samples from two patients were used. Total 16,930 cells were analyzed. The UMAP plots of main cell types for muscles originated from concave and convex side were shown. FAPs indicated fibro/adipogenic progenitors; ECs indicated epithelia cells; SMCs indicated smooth muscle cells; MuSCs/progenitors indicated muscle stem/progenitor cells. **d** Dot plot of highly expressed genes in each cell type. The size of dots indicated the percentage of cells expressing the designated genes. The color of dots represented gene expression level. **e** Violin plot of highly expressed genes in each cell type. **f** Percentage of each cell type at the concave and convex side. **g** UMAP plots of genes marked muscle stem/progenitor cells. **h** Pathway enrichment analysis of differentially expressed genes in muscle stem/progenitor cells located at the concave and convex side of AIS patients. Red bars indicated the enriched terms up-regulated in muscle stem/progenitor cells located at the concave side. Blue bars indicated the enriched terms down-regulated in muscle stem/progenitor cells located at the concave side. The enriched terms related to myogenesis were highlighted by the red solid frames and the terms related to estrogen signaling were highlighted by the red dashed frames. **i** Dot graph of *ESR1* and *ESR2* expression in each cell types identified in muscle located at the concave and convex side of AIS patients, respectively. **j** RT-qPCR analysis of *ESR1* and *ESR2* gene expression levels in muscle stem/progenitor cells isolated from para-spinal muscles derived from non-scoliosis (NS, *n* = 5), congenital scoliosis (CS, *n* = 8) and adolescent idiopathic scoliosis (AIS, *n* = 25) patients. Error bars indicated standard deviation. **P < 0.01; ns indicated no significant changes. **k** ELISA analysis of ESR1 level in muscle stem/progenitor cells isolated from para-spinal muscles derived from non-scoliosis (NS, *n* = 5), congenital scoliosis (CS, *n* = 8) and adolescent idiopathic scoliosis (AIS, *n* = 25) patients. Error bars indicated standard deviation. **P < 0.01; ns indicated no significant changes.

To explore the functions of para-spinal skeletal muscles in AIS, discarded bilateral para-spinal muscle samples during surgery were collected from age-matched female patients with AIS, congenital scoliosis (CS), and no scoliosis (NS, spinal trauma or tumor). The myofiber size from the concave side muscle was smaller compared to that of the convex side in AIS patients (Fig. 1a, b). To further analyze the features of muscle cells in the concave and convex sides, single-cell RNA sequencing (scRNA-seq) was performed. The cells were categorized to 13 groups (Fig. 1c). The typical molecules marking each cell type were listed in Fig. 1d, e. The numbers of macrophages, a subgroup of fibro/adipogenic progenitors (FAPs), muscle stem/progenitor cells, and vein epithelial cells were decreased (Fig. 1f). We paid more attention to the muscle stem/progenitor cells. These cells highly expressed *PAX7* and *MYF5*. *MYOD1* was also expressed (Fig. 1g). Further pathway enrichment analysis of the muscle stem/progenitor cells revealed that ESR signaling was down-regulated in the muscle stem/progenitor cells located at the concave side (Fig. 1h). Consistent with the pathway enrichment analysis, *ESR1* expression was down-regulated in muscle stem/progenitor cells located at the concave side (Fig. 1i). In contrast, the expression of *ESR2* remained to be similar at both side of the spine (Fig. 1i).

We therefore tested the expression of *ESR* in muscle stem/progenitor cells isolated from both the concave and convex side by RT-qPCR. Muscle stem/progenitor (myoblast) cells were isolated from the bilateral para-spinal muscle by FACS as described (Supplementary Fig. S1a–c)^30^. Consistent with the single-cell sequencing analysis data, the mRNA level of *ESR1* was down-regulated in muscle stem/progenitor cells at the concave side when compared to that at the convex side in AIS, while the expression of *ESR2*, another *ESR* isoform, was not changed (Fig. 1j). Consistently, the protein level of ESR1 also decreased in muscle stem/progenitor cells located at the concave side as indicated by ELISA and immunoblotting assays (Fig. 1k; Supplementary Fig. S1d). In contrast, para-spinal muscle stem/progenitor cells from CS and NS did not display the asymmetric expression of ESR1 (Fig. 1j, k), suggesting a potential link between asymmetric expression of ESR1 and AIS.

Taken together, these results reveal the asymmetric expression of ESR1 in para-spinal muscle stem/progenitor cells in AIS patients.

### ESR1 is required for muscle stem/progenitor cell differentiation

Human primary muscle stem/progenitor cells were isolated from the concave side and cultured for 7 days in vitro. The majority of the cells remained PAX7^+^ after 7 days of culturing (Supplementary Fig. S2a, b). The expression level of *ESR1* in cells isolated from the concave side remained low after in vitro culturing (Supplementary Fig. S2c), suggesting the cell intrinsic changes.

**Fig. 2:**
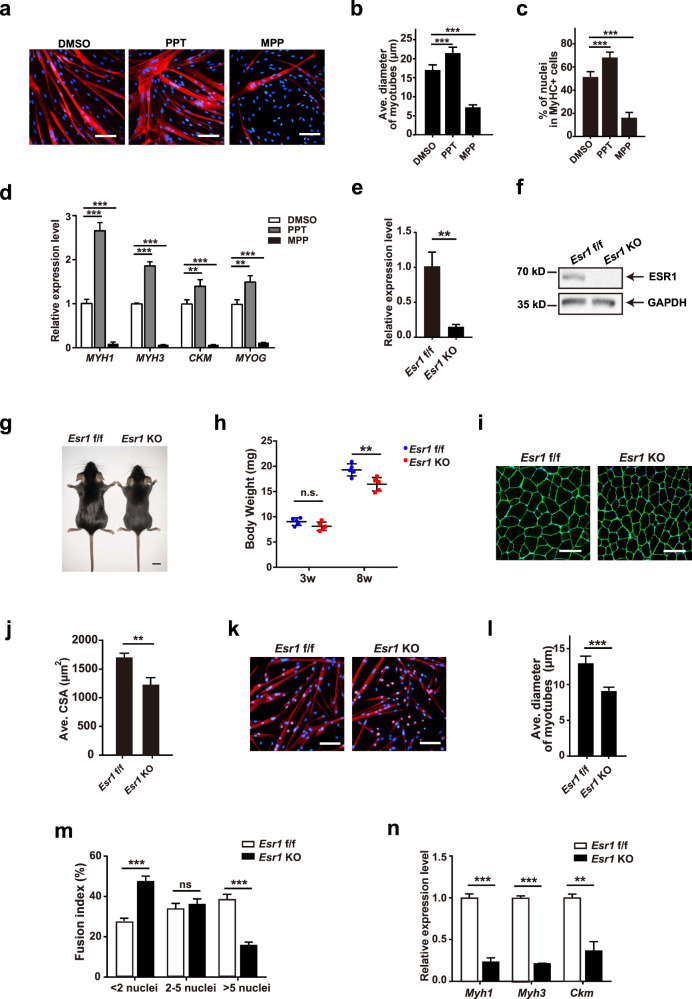
ESR1 is required for muscle stem/progenitor cell differentiation. **a** Representative immunofluorescent staining of differentiating human muscle stem/progenitor cells treated with DMSO, ESR1 agonist PPT, or ESR1 antagonist MPP for six days. Red indicated MyHC; Blue indicated DAPI staining of nuclei. The merged images were shown. Scale bars: 100 μm. **b** Statistical analysis of average diameters of myotubes. Error bars indicated standard deviation (*n* = 5). ***P < 0.001. **c** Statistical analysis of differentiation efficiency. Error bars indicated standard deviation (*n* = 5). ***P < 0.001. **d** Expression levels of differentiation markers. Total RNA was extracted from differentiating cells treated by DMSO, ESR1 agonist PPT, or ESR1 antagonist MPP for six days followed by RT-qPCR analysis. Error bars indicated standard deviation (*n* = 3). ***P < 0.001, **P < 0.01. **e** Gene expression level of *Esr1* in *Esr1* KO muscle stem cells. The primary muscle stem cells were isolated from *Pax7-CreERT2; Esr1*^*f/f*^ mice after tamoxifen induction. *Esr1* expression level was next analyzed by RT-qPCR. Error bars indicated standard deviation (*n* = 3). **P < 0.01. **f** Immunoblotting of Esr1 in *Esr1* KO muscle stem cells. The primary muscle stem cells were isolated from *Pax7-CreERT2; Esr1*^*f/f*^ mice after tamoxifen induction. GAPDH was served as control. **g** Pictures of 8-weeek-old *Esr1*^f/f^ and *Esr1* KO female mice. Scale bar: 1 cm. **h** Statistical analysis of the body weight of *Esr1*^f/f^ and *Esr1* KO mice. Mice were treated with vehicle (*Esr1*^f/f^) or tamoxifen (*Esr1* KO) every other day from 2 weeks old to 3 weeks old. Five female mice were weighted at 3 weeks old and 8 weeks old in each group. Error bars indicated standard deviation (*n* = 5). **P < 0.01; ns indicated no significant changes. **i** Representative immunofluorescent staining of cryosection of para-spinal muscle derived from *Esr1*^f/f^ and *Esr1* KO mice, respectively. Green indicated laminin; Blue indicated DAPI staining of nuclei. The merged images were shown. Scale bars: 100 μm. **j** Statistical analysis of cross-sectional area of para-spinal muscle derived from *Esr1*^f/f^ and *Esr1* KO mice, respectively. At least 500 fibers were analyzed for each sample. Error bars indicated standard deviation (*n* = 4). **P < 0.01. **k** Representative immunofluorescent staining of myotubes differentiated from muscle stem cells isolated from *Esr1*^f/f^ and *Esr1* KO mice, respectively. Red indicated MyHC; Blue indicated DAPI staining of nuclei. The merged images were shown. Scale bars: 100 μm. **l** Statistical analysis of the average diameters of myotubes differentiated from *Esr1*^f/f^ or *Esr1* KO muscle stem cells. Error bars indicated standard deviation (*n* = 5). ***P < 0.001. **m** Fusion index of myotubes differentiated from *Esr1*^f/f^ or *Esr1* KO muscle stem cells. Error bars indicated standard deviation (*n* = 5). ***P < 0.001; ns indicated no significant changes. Fusion index of myotubes differentiated from *Esr1*^f/f^ or *Esr1* KO muscle stem cells. Error bars indicated standard deviation (*n* = 5). ***P < 0.001; ns indicated no significant changes. **n** Expression levels of differentiation markers in myotubes differentiated from *Esr1*^f/f^ or *Esr1* KO muscle stem cells. Error bars indicated standard deviation (*n* = 3). **P < 0.01; ***P < 0.001.

To investigate the function of ESR1 in muscle stem/progenitor cells, the freshly isolated human muscle stem/progenitor cells from no scoliosis participants were treated with ESR1 agonist 4,4′,4″-(4-Propyl-[1H]-pyrazole-1,3,5-triyl) trisphenol (PPT) and ESR1 antagonist 1,3-Bis(4-hydroxyphenyl)-4-methyl-5-[4-(2-piperidinylethoxy)phenol]-1H-pyrazole dihydrochloride (MPP), respectively. Cell proliferation was not affected by the treatment as shown by EdU labeling experiments (Supplementary Fig. S2d, e). The function of ESR1 in myogenic differentiation was next studied. PPT or MPP was added when human muscle stem/progenitor cells were induced to differentiate, and the cells were harvested at day 1, 3, and 6 post differentiation to evaluate the differentiation process. The size of myotubes and differentiation efficiency increased after ESR1 agonist PPT treatment (Fig. 2a–c; Supplementary Fig. S2f–m). Consistently, the expression levels of differentiation related genes such as *MYH1*, *MYH3*, *MYOG*, and *CKM* were elevated (Fig. 2d; Supplementary Fig. S2i, m), suggesting improvement of muscle stem/progenitor cell differentiation. In contrast, differentiation efficiency decreased after ESR1 antagonist MPP treatment (Fig. 2a–d; Supplementary Fig. S2f–m). These results suggest that activation of ESR1 signaling was required for human muscle stem/progenitor cell differentiation.

ESR1 knockdown experiments in human stem/progenitor cells were then performed to further confirm its functions. ESR1 expression was efficiently knocked down in human muscle stem/progenitor cells after RNAi (Supplementary Fig. S2n, o). Differentiation defects were observed as indicated by reduced myotube size (Supplementary Fig. S2p, q), lower differentiation efficiency (Supplementary Fig. S2r), and reduced expression of differentiation markers *MYH1*, *MYH3*, and *CKM* (Supplementary Fig. S2s).

Muscle stem cell specific *Esr1* knockout (KO) mice were obtained after intraperitoneal (i.p.) tamoxifen injection in *Pax7-CreERT2; Esr1*^*f/f*^ mice. These mice were treated with oil vehicle or tamoxifen (*Esr1* KO) every other day for one week (from 2 weeks old to 3 weeks old). Muscle stem cells were isolated as previously described^31^, and the knockout of Esr1 was confirmed (Fig. 2e, f). The *Esr1* KO mice displayed smaller body size and lower body weight at 8 weeks old, although these mice were born with normal weight and remained normal until 3 weeks old (Fig. 2g, h). Consistently, the para-spinal myofiber size of the *Esr1* KO mice was smaller (Fig. 2i, j). *Esr1* KO muscle stem cells were isolated and displayed differentiation defects (Fig. 2k–n). Together, these results suggest that ESR1 is critical for the adolescent skeletal muscle growth by regulating muscle stem cell differentiation in both human and mouse.

### ESR1 activates AKT signaling in human muscle stem/progenitor cells

**Fig. 3:**
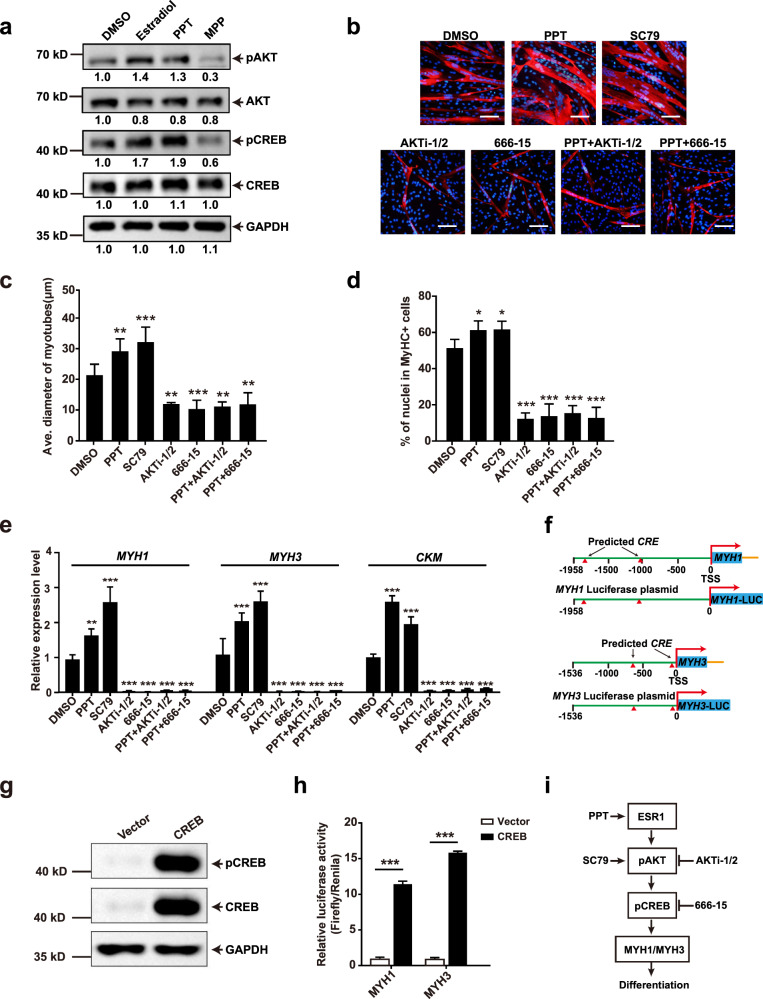
ESR1 activates AKT signaling in human muscle stem/progenitor cells. **a** Representative immunoblotting of phosphorylated AKT, total AKT, phosphorylated CREB, total CREB, and GAPDH of human muscle stem/progenitor cells with different treatments. Upon initiation of differentiation, DMSO, estradiol, ESR1 agonist PPT, or ESR1 antagonist MPP was added in culture, respectively. Cells were treated for 2 h and harvested for analysis. Total proteins were extracted and subjected for immunoblotting. The quantification of the band intensity was listed below each band. **b** Representative immunofluorescent staining of MyHC with differentiated human muscle stem/progenitor cells. Human muscle stem/progenitor cells were differentiated for 6 days and treated with DMSO, PPT, SC79, AKTi-1/2, 666-15, PPT combined with AKTi-1/2, and PPT combined with 666-15, respectively. PPT, ESR1 agonist; MPP, ESR1 antagonist; SC79, AKT signaling agonist; AKTi-1/2, AKT signaling inhibitor; 666-15, CREB inhibitor. Red indicated MyHC; blue indicated DAPI staining of nuclei. The merged images were shown. Scale bars: 100 μm. **c** Statistical analysis of the average diameter of myotubes by small molecule treatment. Error bars indicated standard deviation (*n* = 5). **P < 0.01; ***P < 0.001. **d** Statistical analysis of the differentiation efficiency. Error bars indicated standard deviation (*n* = 5). *P < 0.05; ***P < 0.001. **e** Expression levels of *MYH1*, *MYH3*, and *CKM*. Human muscle stem/progenitor cells were induced to differentiate for 6 days while treating with various types of small molecules targeting ESR1-AKT-CREB signaling. Total RNA was extracted from each sample and subjected for RT-qPCR analysis. Error bars indicated standard deviation (*n* = 3). **P < 0.01; ***P < 0.001. **f** The scheme of luciferase construct. The red triangles indicated predicted CREB binding site (cAMP response element). TSS indicated the transcription starting site. The potential promoter region of *MYH1* (–1958 bp to TSS) or *MYH3* (–1536 bp to TSS) was cloned into the *MYH*-Luciferase plasmid. **g** Expression of ectopic CREB and phosphorylated CREB in luciferase assays. The 293T cells were transfected by plasmid encoding CREB and harvested 2 days after transfection. Total proteins were extracted and subjected for immunoblotting of CREB and phosphorylated CREB. GAPDH served as the internal control. **h** Statistical analysis of luciferase essays. Error bars indicated standard deviation (*n* = 3). ***P < 0.001. **i** ESR1 signaling regulated muscle stem cell differentiation. PPT, ESR1 agonist; SC79, AKT signaling agonist; AKTi-1/2, AKT signaling inhibitor; 666-15, CREB inhibitor.

It has been reported that estradiol activates AKT signaling in many rodent cell types^32,33^. AKT signaling is a well-studied pathway activating muscle differentiation^34^. We therefore tested whether activation of ESR1 can also stimulate AKT signaling in human muscle stem/progenitor cells. Healthy human muscle stem/progenitor cells were treated with estradiol, ESR1 agonist PPT, and ESR1 antagonist MPP upon differentiation induction, respectively. The level of phosphorylated AKT elevated after estradiol and PPT treatment, while MPP treatment led to the decline of phosphorylated AKT level (Fig. 3a). Consistently, phosphorylation of CREB, which has been reported previously to be the downstream target of AKT signaling^35,36^, was also elevated upon estradiol and PPT treatments and decreased upon MPP treatment (Fig. 3a). These results suggest that activation of ESR1 stimulates AKT signaling in human muscle stem/progenitor cells.

AKT agonist SC79 displayed the similar differentiation improvement effects as ESR1 agonist PPT (Fig. 3b–e). Differentiation defects were observed when the primary human muscle stem/progenitor cells were treated with AKT inhibitor AKTi-1/2 or CREB inhibitor 666-15, phenocopying that of MPP (Fig. 3b–e). Activation of ESR1 by PPT was unable to rescue the differentiation defects caused by inhibition of AKT or CREB (Fig. 3b–e), suggesting that AKT is downstream of ESR1. Promoter sequence analysis demonstrated the presence of CREB recognition elements (CRE) in the promoter regions of *MYH1* and *MYH3*. To confirm the activation of *MYH1* and *MYH3* by CREB, the luciferase reporter assays were performed using construct containing firefly luciferase driven by the promoter of target gene (Fig. 3f), and renilla luciferase served as the internal control. Overexpression of CREB increased the luciferase activity (Fig. 3g, h), suggesting that CREB is capable of activating the transcription of *MYH1* and *MYH3* genes and promoting differentiation.

Altogether, these results reveal a ESR1-AKT-CREB-MYH axis of signaling cascade promoting human muscle stem/progenitor cell differentiation (Fig. 3i).

### Reduced ESR1 signaling activity in human muscle stem/progenitor cells at the concave side in AIS patients

Since the human muscle stem/progenitor cells at the concave side of AIS displayed reduced ESR1 expression, we further investigated whether the ESR1-AKT-CREB-MYH signaling cascade was also repressed.

The primary human muscle stem/progenitor cells were isolated from the bilateral para-spinal muscles and treated with ESR1 agonist PPT. As expected, PPT treatment activated the phosphorylation of AKT and CREB followed by the elevated expression of MyHC (Supplementary Fig. S3a). When human muscle stem/progenitor cells were treated by estradiol or PPT, both treatments improved the differentiation of human muscle stem/progenitor cells (Supplementary Fig. S3b, c). The cells from concave side showed less improvement of differentiation ability after estradiol and PPT treatments when compared with the convex cells (Supplementary Fig. S3d–g). Consistently, although the phosphorylated AKT and CREB levels were elevated after ESR1 or PPT treatment for both sides, the concave cells were also less increased (Supplementary Fig. S3f). Furthermore, the levels of phosphorylated AKT and CREB were down-regulated in human muscle stem/progenitor cells from the concave side. Consistently, the MyHC expression level also decreased in the concave human muscle stem/progenitor cells after differentiation induction (Supplementary Fig. S3g). In contrast, the muscle tissue, which contains mainly the muscle fibers, from both convex and concave sides did not show any obvious differences on the level of phosphorylated AKT, phosphorylated CREB, and MyHC, suggesting that the AKT-CREB-MyHC signaling is specifically inactivated in muscle stem/progenitor cells at the concave side, not in the differentiated myofibers. Taking together, these results suggest that human muscle stem/progenitor cells at the concave side display reduced ESR1-AKT-CREB signaling activity and impaired differentiation ability.

### Asymmetric inactivation of ESR1 in para-spinal muscle leads to scoliosis

We next went on to test whether the inactivation of ESR1 signaling cascade increased the susceptibility to scoliosis in vivo using mouse model. We first tested whether the similar effects of PPT and MPP in human muscle stem/progenitor cells can also be recapitulated in mouse muscle stem cells. Mouse muscle stem cells were isolated as described^31^ and PPT improved the myogenic differentiation, while MPP significantly inhibited differentiation (Supplementary Fig. S4a–d). These data indicate that ESR1 antagonist MPP could impair the differentiation ability of mouse muscle stem cells. We then investigated whether muscle strength was impaired in muscle stem cell-specific *Esr1*-KO mice. Muscle stem cell specific *Esr1*-KO mice were obtained after intraperitoneal tamoxifen injection in *Pax7-CreERT2; Esr1*^*f/f*^ *mice*. These mice were treated with oil vehicle (*Esr1*^*f/f*^) or tamoxifen (*Esr1* KO) every other day for one week (from 2 weeks old to 3 weeks old). Although tibialis anterior muscle strength in *Esr1* KO mice remained normal at 3 weeks old (Supplementary Fig. S4e), the mice displayed significantly deceased muscle strength at 8 weeks old (Supplementary Fig. S4f). Thus, asymmetric *Esr1* expression might contribute to asymmetric biomechanical load from para-spinal muscle.

**Fig. 4:**
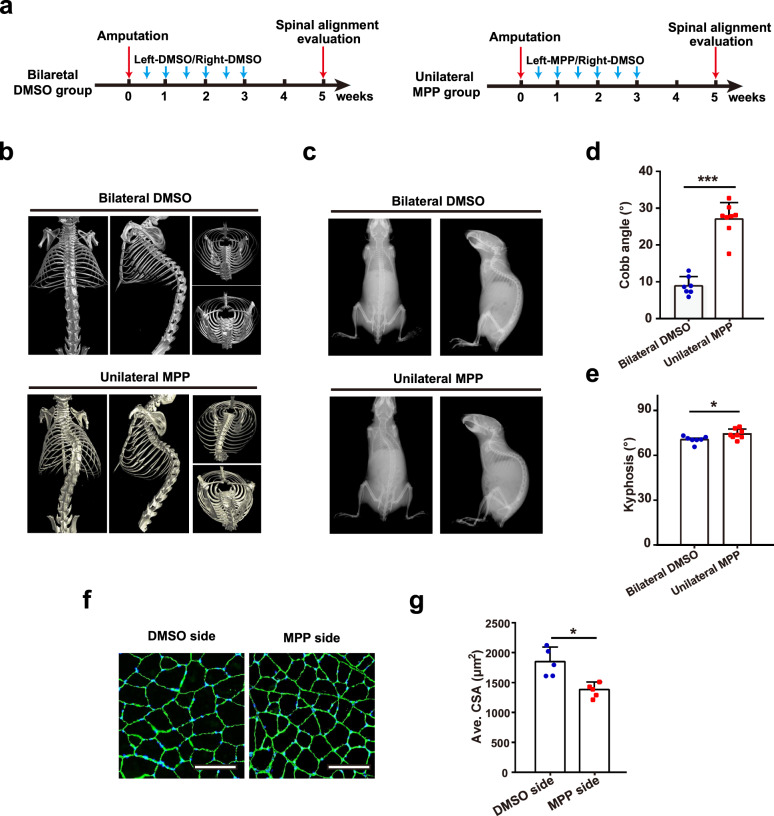
Asymmetric inactivation of ESR1 in para-spinal muscle leads to scoliosis. **a** Scheme of unilateral MPP treatment. Bipedal mice of 3 weeks old were subjected for unilateral intramuscular injection of MPP at the left side of the para-spinal muscle, while DMSO was injected to the opposite side. Continuous injections were performed twice every week for 3 weeks. The spinal alignment was evaluated 5 weeks after the first MPP injection. **b** Micro-CT images for spinal alignment and thoracic cage symmetry evaluation in bilateral DMSO and unilateral MPP group 5 weeks after the first MPP injection. **c** In vivo X-ray images of spinal alignment in bilateral DMSO and unilateral MPP group 5 weeks after the first MPP injection. **d** Statistical analysis of Cobb angle in coronal plane for bilateral DMSO group (*n* = 7) and unilateral MPP group (*n* = 8) 5 weeks after the first injection. Error bars indicated standard deviation. ***P < 0.001. **e** Statistical analysis of kyphosis in sagittal plane for bilateral DMSO group (*n* = 7) and unilateral MPP group (*n* = 8) 5 weeks after the first injection. Error bars indicated standard deviation. *P < 0.05. **f** Representative immunofluorescent staining of para-spinal muscle sections from unilateral MPP group. Cryosections were obtained from the para-spinal muscles isolated from the DMSO injection side and MPP injection side. The cryosections were stained with anti-Laminin antibody. Green indicated laminin; Blue indicated DAPI staining of nuclei. The merged images were shown. Scale bars: 100 μm. **g** Statistical analysis of the CSA of myofibers from unilateral MPP group. At least 400 fibers were analyzed for each sample. Error bars indicated standard deviation (*n* = 5). *P < 0.05.

We next investigated whether asymmetric inactivation of ESR1 signaling in para-spinal muscles could increase the susceptibility to scoliosis in a mouse model. Bipedal mouse model was generated using 3-week-old female mice. ESR1 antagonist MPP was injected to the left side of the para-spinal muscles, while DMSO was injected to the opposite side (Fig. 4a). Continuous injections were performed twice every week for 3 weeks to mimic the unilaterally reduced ESR1 signaling in AIS patients. The spinal alignment was evaluated by micro-CT and X-Ray 2 weeks after the final MPP injection (Fig. 4a). Compared to the group with bilateral DMSO injection, more severe spinal misalignment in both coronal and sagittal planes was observed in unilateral MPP injection group (Fig. 4b–e). In addition, unilateral MPP injection group displayed asymmetric thoracic cage (Fig. 4b). The mice were then sacrificed and the para-spinal muscles at both sides were harvested for further examination. The size of myofibers at the side with MPP injection was smaller compared to that at the DMSO injection side (Fig. 4f, g). Taken together, these results suggest that inactivation of ESR1 signaling asymmetrically in the para-spinal muscles increases the susceptibility to scoliosis in vivo.

### Reactivation of ESR1-AKT-CREB-MYH signaling cascade rescues the differentiation defects of muscle stem/progenitor cells at the concave side

To confirm the functions of reactivation of ESR1-AKT-CREB signaling in human muscle stem/progenitor cells, ESR1 or CREB was overexpressed by infecting human muscle stem/progenitor cells isolated from the concave side with adenovirus encoding *ESR1* or *CREB* (Supplementary Fig. S5a). Then differentiation assay was performed in differentiation medium with estradiol supplementation. The myotube size, differentiation efficiency, and the expression levels of differentiation markers were rescued to the similar level of myotubes differentiated from muscle stem/progenitor cells isolated from the convex side (Supplementary Fig. S5a–d). These results suggested that reactivation of ESR1-AKT-CREB-MYH axis of signaling cascade could rescue the differentiation defects of the human muscle stem/progenitor cells located at the concave side.

**Fig. 5:**
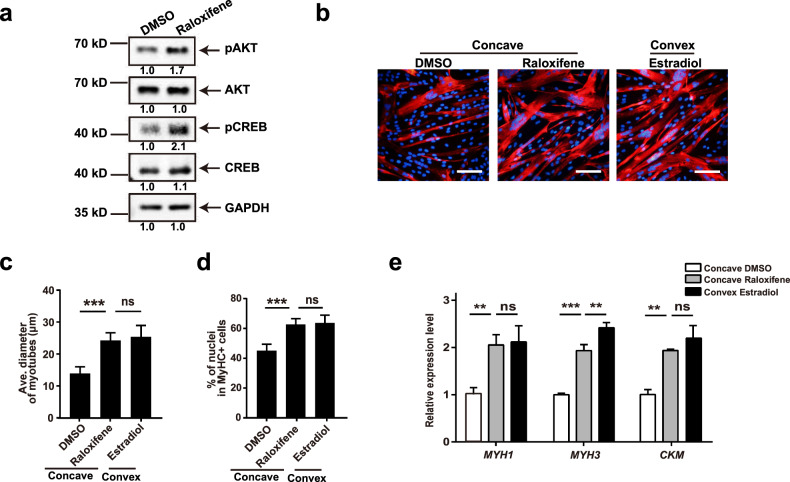
Raloxifene activates ESR1-AKT-CREB-MYH signaling and rescues the differentiation defects of concave muscle stem/progenitor cells. **a** Representative immunoblotting of AKT, phosphorylated AKT, CREB, and phosphorylated CREB of human muscle stem/progenitor cells. Upon initiation of differentiation, DMSO or Raloxifene was added in culture for 2 h. Then total proteins were extracted and subjected for immunoblotting. GAPDH served as an internal control. The quantification of the band intensity was listed below each band. **b** Representative immunofluorescent staining of human muscle stem/progenitor cells treated with Raloxifene. Human muscle stem/progenitor cells were isolated from either the convex or concave side of AIS patients. The cells isolated from the concave side (treated by DMSO or Raloxifene) and convex side (treated by estradiol) were differentiated for 6 days and subjected for MyHC immunofluorescent staining. Red indicated MyHC; blue indicated DAPI staining of nuclei. The merged images were shown. Scale bars: 100 μm. **c** The statistical analysis of the average diameter of myotubes differentiated from bilateral muscle stem/progenitor cells in AIS with different treatments. Error bars indicated standard deviation (*n* = 5). ***P < 0.001; ns indicated no significant changes. **d** The statistical analysis of the differentiation efficiency after different treatments. Error bars indicated standard deviation (*n* = 5). ***P < 0.001; ns indicated no significant changes. **e** The mRNA level of *MYH1*, *MYH3*, and *CKM* in myotubes with different treatments. Human muscle stem/progenitor cells isolated from the concave side (treated by DMSO or Raloxifene) and convex side (treated by estradiol) were differentiated for 6 days. Total RNA was extracted and subjected for RT-qPCR analysis. Error bars indicated standard deviation (*n* = 3). **P < 0.01, ***P < 0.001; ns indicated no significant changes.

To find the molecules capable of increasing the muscle functions at the concave side, we screened the FDA approved estrogen receptor modulators to test their abilities to activate ESR1-AKT-CREB-MYH signaling. Raloxifene is an FDA approved selective estrogen receptor modulator mainly to treat osteoporosis^37^. It can efficiently activate AKT and CREB in human muscle stem/progenitor cells (Fig. 5a). Furthermore, Raloxifene treatment rescued the differentiation defects of human muscle stem/progenitor cells at the concave side (Fig. 5b–e). These results suggest that Raloxifene could be a potential drug to treat AIS by efficiently activating ESR1-AKT-CREB-MYH signaling.

### Raloxifene alleviates the progression of scoliosis

Since Raloxifene rescued the differentiation defects of muscle stem/progenitor cells isolated from the concave side, we therefore explored the possibility to treat scoliosis by Raloxifene in vivo.

**Fig. 6:**
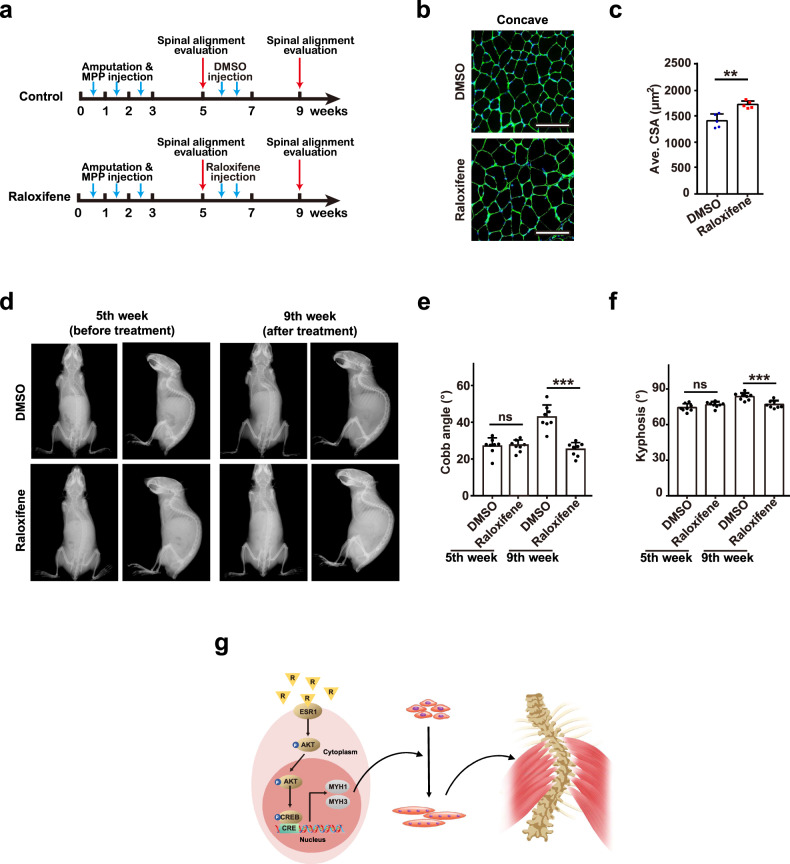
Raloxifene alleviates the progression of scoliosis. **a** Scheme of Raloxifene treatment in AIS animal model. Scoliosis mouse model was generated as aforementioned. After the occurrence of scoliosis, intramuscular Raloxifene injections were performed at the concave para-spinal muscle once every 3.5 days for 2 weeks (from the 5th week to the 7th week). The spinal alignment and para-spinal muscle size were then analyzed at 9th week. **b** Representative immunofluorescent staining of Raloxifene-treated or DMSO-treated concave para-spinal muscle 2 weeks after final injection. Cryosections were obtained from para-spinal muscle at the concave side with either DMSO mock treatment or Raloxifene treatment and subjected for laminin immunofluorescent staining. Green indicated laminin; blue indicated DAPI staining of nuclei. The merged images were shown. Scale bars: 100 μm. **c** Statistical analysis of the CSA of myofibers in Raloxifene treated or DMSO treated concave para-spinal muscle. At least 500 fibers were analyzed for each sample. Error bars indicated standard deviation (*n* = 5). **P < 0.01. **d** In vivo X-ray images of spinal alignment before and after Raloxifene treatment. **e** Statistical analysis of Cobb angle in coronal plane after DMSO mock treatment (*n* = 8) and Raloxifene treatment (*n* = 8), respectively. 5th week indicated before treatment; 9th week indicated after treatment. Error bars indicated standard deviation (*n* = 8). ns indicated no significant changes; ***P < 0.001. **f** Statistical analysis of kyphosis in sagittal plane after DMSO mock treatment (*n* = 8) and Raloxifene treatment (*n* = 8), respectively. 5th week indicated before treatment; 9th week indicated after treatment. Error bars indicated standard deviation (*n* = 8). ns indicated no significant changes; ***P < 0.001. **g** Asymmetric ESR1 signaling increased the susceptibility to AIS. Restoration of ESR1 signaling by Raloxifene (indicated by R in yellow triangles) alleviated the progression of scoliosis.

Scoliosis mouse model was generated by unilateral MPP injection as described above. Raloxifene was injected to the concave para-spinal muscle once every 3.5 days for 2 weeks, while DMSO was injected into the contralateral para-spinal muscles. In control group, DMSO was injected to bilateral para-spinal muscle once every 3.5 days for 2 weeks (Fig. 6a). The Raloxifene treated mice were sacrificed and the bilateral para-spinal muscles were harvested for further analysis. In the Raloxifene treated concave para-spinal muscle, myofiber size increased when compared to that in the control group (Fig. 6b, c). More importantly, after Raloxifene treatment, the spinal alignment was improved (Fig. 6d). And the coronal curve and kyphosis progression were alleviated compared to those in the control group (Fig. 6e, f), suggesting that Raloxifene slowed down the progression of scoliosis. These results suggest that Raloxifene could be the potential medicine to treat AIS.

## Discussion

Our results established the link between adolescent myogenesis and the initiation and development of AIS. The abnormal myogenesis caused by the disruption of the ESR1 signaling in muscle stem/progenitor cells at one side of the para-spinal muscle leads to AIS. Furthermore, efficient reactivation of ESR1-AKT-CREB-MYH signaling improves the differentiation ability of the muscle stem/progenitor cells at the concave side. Correction of the imbalance of ESR1-AKT-CREB-MYH signaling between two sides of para-spinal muscle by the FDA approved medicine Raloxifene mitigates the progression of AIS (Fig. 6g). Our work provides a paradigm of para-spinal muscle development defects leading to AIS and identifies a new target tissue to treat AIS. It suggests that Raloxifene may be a promising therapeutic drug to treat AIS patients who have imbalanced ESR1 signaling in the para-spinal muscles.

One of the important findings in the current study is that para-spinal muscle could be a primary cause for AIS. Many clinical observations suggest that unbalanced para-vertebral muscle function may attribute to the initiation and development of AIS^16,19^. The balanced myogenesis supported by muscle stem cells at the bilateral para-vertebral muscle contributes greatly to the symmetrical growth of para-spinal muscle. However, these links were only supported by clinical correlations and other peripheral evidence. Here we provide direct a link between myogenesis defects caused by the irregulated ERS1 signaling in muscle stem cells and the initiation and development of AIS. AIS usually occurs in puberty, coincident with a wave of muscle mass gain from myogenesis, hinting a potential link between myogenesis defects and AIS. Although the asymmetry of the para-spinal muscles in AIS has been reported previously^14,16,19,20^, whether it is the secondary effect of scoliosis or a major causative factor for scoliosis has not been truly determined. In current study, age-matched non-scoliosis group and congenital scoliosis group were involved for strict control. No significant difference of ESR1 expression in muscle stem/progenitor cells was identified between bilateral sides in congenital scoliosis or non-scoliosis group, while there was significantly decreased expression level of ESR1 on concave side in AIS patients. Decreased ESR1 impaired the differentiation of concave muscle stem/progenitor cells, and thus contributing to the initiation and progression of AIS. These results suggest that the para-spinal muscle may be a major causative factor for AIS and reveal a new target organ to develop treatment for AIS.

Due to the disproportionally high incidence of AIS in females, estrogen has long been suspected to have a role in the initiation of AIS^38–40^. However, no consistent results have been obtained while measuring the circulating estrogen level in female AIS patients^38–40^, leaving the link between estrogen and AIS obscure. Studies have found that the polymorphisms of Estrogen Receptor (ESR) related to the susceptibility of AIS^41,42^. Recent studies also showed different methylation levels of *ESR* in para-spinal muscle, which may be associated with curve severity^43,44^. These evidence indicates the potential relationship between ESR and AIS. However, the direct link between ESR and AIS and the mechanism have not been revealed yet.

Adolescent females are highly susceptible to AIS^3^. However, no consistent changes of the circulating estrogen levels in adolescent female patients have been identified^38–40^. Here we found that the expression of ESR1 is a node in estrogen signaling being hit in AIS patient. There are limited studies investigating the function of ESR1 in muscle stem cells^32,45,46^, while contradictory conclusions were obtained for the role of ESR1 in myogenic differentiation of mouse and rat myoblast cells^32,46^. Its function on human muscle stem/progenitor cells still remains unknown before the current investigation, especially for adolescent females with dramatic change of hormone levels. Thus, for the first time, the current study reveals that ESR1 is critical for differentiation of muscle stem/progenitor cells in adolescent females.

Further study should be conducted to investigate the observed asymmetric ESR1 expression of para-spinal muscle stem/progenitor cells. According to the previous research, asymmetric ESR1 level of para-spinal muscle stem cells in AIS is more likely to be caused by differential DNA methylation. Firstly, there are tissue-dependent and differentially methylated regions (T-DMRs) in *ESR1*^47^. T-DMR is a DNA methylation region which could regulate gene transcriptional activity, and it has been demonstrated that aberrant DNA methylation of the T-DMR was associated with the impaired expression of ESR1 in ovarian endometrioma^48^. In addition, a recent study revealed that DNA methylation levels in *ESR1* T-DMR from concave para-spinal muscle tissue were associated with curve severity^43^. They also indicated that the DNA methylation patterns of *ESR1* T-DMRs depended on the cell localization in skeletal muscle tissue^43^. Thus, the asymmetric ESR1 level of para-spinal muscle stem cells in AIS could be resulted from asymmetric DNA methylation level in T-DMRs, after an initial epigenetic trigger event.

Conservative treatment was always recommended for AIS with Cobb angle less than 40 degrees, while functional exercise and bracing are routinely performed^49,50^. However, the long treatment course and time-consuming conservative treatment strategy frequently contribute to unsatisfactory medical compliance. Drug therapy might provide an easier and practical strategy to slow down the curve progression. Raloxifene is a popular selective estrogen receptor modulator to treat postmenopausal osteoporosis and breast cancer^4,37^. The usage, safety, and feasibility of Raloxifene for AIS is an interesting question for further exploration. Taking the conventional FDA approved medicine Raloxifene to a new use to treat AIS will facilitate the development of new treatments for AIS.

## Methods

### Animals

All experiments were conducted in female mice maintained in specific pathogen-free animal facility in individually ventilated cages with controlled temperature (22 ± 1°C) and light (12 h light/dark cycle). Animal care and use were in accordance with the guidelines of the institution. The study protocol has been approved by ethics committee of local institution (Approval No. XHEC-F-2021-012). *Pax7-CreERT2* mice were purchased from Jackson Laboratory, and *Esr1*^*f/f*^ mice were purchased from Gem Pharmatech. Muscle stem cell specific gene knockout was induced by i.p. injection of 100 μL of 10 mg/mL tamoxifen (ABCONE, cat#T56488) every 2 days for a week.

Bipedal mice were established to simulate upright posture as described previously^51^. Briefly, forelimb and tail of mice were firstly removed at 3 weeks old. With progressively raising water and food, these bipedal mice will gradually maintain standing posture. Unilateral intramuscular injection of 100 μL 100 nM MPP (Tocris, cat#1991) at the left side of the para-spinal muscle was performed in 3-week-old mice twice every week for 3 weeks.

In the scoliosis mouse model, intramuscular injections of 10 nM Raloxifene (Tocris, cat#2280) were performed at the concave para-spinal muscle once every 3.5 days for 2 weeks. The spinal alignment and para-spinal muscle size were then analyzed at 2 weeks after raloxifene injection.

### Human samples

Discarded bilateral para-spinal muscles during surgery from level of apical vertebra (for scoliosis patients) or similar vertebral level region (non-scoliosis control) were collected as described previously^28,29,52^. There was no additional risk for patients during harvesting procedure. The participants were females from 10 to 18 years old. The study was approved by the ethics committee of local institution (Approval No. XHEC-D-2019-093) and the signed informed consents were obtained from all participants and their legal guardians.

### Isolation of muscle stem/progenitor cells

Muscle stem/progenitor cells were isolated as described previously^31,53^. Briefly, muscle tissues were sliced to 1 mm^3^ pieces, digested by collagenase II (Worthington biochemical, 700-800 U/mL, cat#LS004177) for an hour and subsequently digested by mixtures of collagenase II and dispase (Life Technologies,11 U/mL, cat#17105-041) for 30 min. The digested mixture was passed 10 times through a 20-gauge needle and filtered through a 40-µm cell strainer (BD Falcon, cat#352340). The erythrocytes were removed by red blood cell lysis (Thermo Fisher Scientific, cat#00-433-57). The human cell suspension was stained with PE-Cy5 anti-human CD45 (BD Pharmingen, cat#555484, 1:25), Percp-Cy5.5 anti-human CD31 (BioLegend, cat#303132, 1:100), AF-488 anti-human CD29 (BioLegend, cat#303016, 1:100) and BV421 anti-human CD56 (BD, cat#562751, 1:100) for 45 min at 4 °C. The mouse cell suspension was stained with a cocktail of APC anti-mouse CD31 (BioLegend, cat#102510, 1:100), APC anti-mouse CD45 (BioLegend, cat#103112, 1:100), FITC anti-mouse Sca1 (BioLegend, cat#108106, 1:100) and Biotin anti-mouse VCAM1 (BioLegend, cat#105703, 1:100) for 45 min at 4 °C. All cell suspensions were washed with PBS and stained with PE/Cy7 Streptavidin (BioLegend, cat#405206, 1:100) for 15 min and sorted by FACS using Aria III or Influx (BD Biosciences).

### Single-cell RNA sequencing

Single-cell RNA sequencing was performed in bilateral paraspinal muscles from two AIS patients. The cell suspension from para-spinal muscle was prepared as described above. Live cells were FACS sorted by Hoechst (Sangon Biotech, cat#A601112) and PI (Sangon Biotech, cat#E607328), washed twice with PBS containing 0.04% BSA, and subjected for library preparasion with Chromium Single Cell 3’ Reagent Kits (10X genomics, cat# 1000121-1000157). The sequencing was performed on Illumina Novaseq 6000 platform (Illumina).

Seurat R (Version 3.2.0) package^54^ was used to analyze the single-cell RNA-seq data. Cells with fewer than 200 genes, or more than 6000 genes, or more than 16% mitochondrial genes were further excluded from the downstream analysis. After quality control, 16,930 cells remained and were used for downstream analysis. Sequencing reads for each gene were normalized to total unique molecular identifiers (UMIs) in each cell to obtain normalized UMI values by “NormalizeData” function. The “ScaleData” function was used to scale and center expression levels in the data set for dimensional reduction. To avoid batch effects among samples, canonical correlation analysis (CCA) was first used to project the data into a subspace to identify correlations across datasets. The mutual nearest neighbors (MNNs) were then computed in the CCA subspace and serve as “anchors” to correct the data. Total cell clustering was performed by “FindClusters” function at a resolution of 0.15, and dimensionality reduction was performed with “RunUMAP” function. We finally clustered the cells into 13 clusters. To identify cell types, the clusters were conducted using “FindClusters” function. Marker genes for each cluster identified with the “FindAllMarkers” function and only those with *P* values < 0.05 and |logFC| > 0.25 were regarded as marker genes. Cell types were identified according to the expression of canonical marker genes.

Differential gene expression analysis was performed with the “FindMarkers” function between convex and concave side patients using the Wilcox test. Only those with *P* value < 0.05 and |logFC| > 0.25 were identified as differential expressed genes. Pathway enrichment analysis of differential expressed genes was performed by clusterProfiler (version 3.18.1)^55^. Dot plot of marker gene expression for each cluster was generated using DotPlot in Seurat package. The same function was also used to plot the expression of *ESR1*/*ESR2* in each cluster for concave and convex side with split.by parameter. Results were visualized with the ggplot2 R package (https://ggplot2.tidyverse.org/). The single cell sequencing data were deposited to Sequence Read Archive (SRA) database with the accession number PRJNA722100.

### Cell culture and differentiation

Primary human muscle stem/progenitor cells were cultured with F10 basal medium (Gibco, cat#11550043) containing 20% FBS (Gibco, cat#10-013-CV), 2.5 ng/mL bFGF (R&D, cat#233-FB-025). Mouse muscle stem cells were cultured with F10 basal medium (Gibco, cat#11550043) containing 20% FBS (Gibco, cat#10-013-CV), 2.5 ng/mL bFGF (R&D, cat#233-FB-025), 5 ng/mL IL-1α (Peprotech, cat#211-11 A), 5 ng/mL IL-13 (Peprotech, cat#210-13), 5 ng/mL IFN-γ (Peprotech, cat#315-05), 5 ng/mL TNF-α (Peprotech, cat#315-01 A), and 1% penicillin-streptomycin (Gibco, cat#15140-122) in collagen-coated dishes at 37 °C in 5% CO_2_ as described previously^31^. The differentiation medium was DMEM (Gibco, cat#11965118) containing 2% horse serum (HyClone, cat#HYCLSH30074.03HI), and 1% penicillin-streptomycin (Gibco, cat#15140-122).

### Immunohistological and immunofluorescent staining

Fresh muscle tissues were mounted in frozen section medium (Thermo Fisher Scientific, cat#6520) and sliced by cryostat (Leica, cat#CM1860) to obtain 10 µm cryosections.

The cryosections or cultured cells were fixed in PBS containing 4% paraformaldehyde (Sigma-Aldrich, cat#30525) for 15 min, permeabilized in 0.5% Triton X-100 for 15 min at room temperature, blocked in PBS containing 1% BSA (Beyotime Biotechnology, cat#ST023). Anti-PAX7 (Developmental Studies Hybridoma Bank, 1:100), anti-Laminin (Abcam, cat#ab11575, 1:500) or anti-MyHC (Millipore, cat#05-716, 1:1000) was applied and incubated overnight at 4 °C. Alexa 488- or Alexa 594-labeled anti-mouse or anti-rabbit secondary antibodies (Invitrogen, 1:1000) were applied to incubate for 1 h at room temperature. The samples were then stained with 1 μg/mL DAPI (Vector Laboratories, cat#H-1200) and finally mounted with antifade mounting media (Vector Laboratories, cat#H-100) for subsequent imaging.

### Measurement of myofibers and myotubes

At least five independent visual fields were randomly selected and evaluated for each sample. Laminin staining was used to identify the boundary of myofibers, and MyHC staining was performed to determine the outline of myotubes. Image J software was used to count cell nuclei (total nuclei and nuclei within myotubes), analyze the diameter of myotubes, and measure the cross-sectional area of myofibers. All images were analyzed and evaluated by investigators in a blinded manner.

### Gene expression analysis

Total RNA was isolated using TRIzol Reagent (Invitrogen, cat#15596-018) according to the manufacturer’s instruction and reverse transcribed by MuLV reverse transcriptase (NEB, cat#M0253L) at 42 °C for 60 min. Quantitative PCR reactions were performed with FastStart Universal SYBR Green Master (Roche, cat#4913914001) in the ABI Q6 real-time PCR system (ABI). *GAPDH* served as the internal control. The primers for RT-qPCR were listed as below:

**Table:** 

Human *GAPDH*-F	5′-CAAGGCTGAGAACGGGAAGC-3′
Human *GAPDH*-R	5′-AGGGGGCAGAGATGATGACC-3′
Human *ESR1*-F	5′-CCCACTCAACAGCGTGTCTC-3′
Human *ESR1*-R	5′-CGTCGATTATCTGAATTTGGCCT-3′
Human *ESR2*-F	5′-AGCACGGCTCCATATACATACC-3′
Human *ESR2*-R	5′-TGGACCACTAAAGGAGAAAGGT-3′
Human *MYH1*-F	5′-GGGAGACCTAAAATTGGCTCAA-3′
Human *MYH1*-R	5′-TTGCAGACCGCTCATTTCAAA-3′
Human *MYH3*-F	5′-ATTGCTTCGTGGTGGACTCAA-3′
Human *MYH3*-R	5′-GGCCATGTCTTCGATCCTGTC-3′
Human *CKM*-F	5′-ATGCCATTCGGTAACACCCAC-3′
Human *CKM*-R	5′-GCTTCTTGTAGAGTTCAAGGGTC-3′
Mouse *Gapdh*-F	5′-ACCCAGAAGACTGTGGATGG-3′
Mouse *Gapdh*-R	5′-ACACATTGGGGGTAGGAACA-3′
Mouse *Esr1*-F	5′-CCCGCCTTCTACAGGTCTAAT-3′
Mouse *Esr1*-R	5′-CTTTCTCGTTACTGCTGGACAG-3′
Mouse *Myh1*-F	5′-GCGAATCGAGGCTCAGAACAA-3′
Mouse *Myh1*-R	5′-GTAGTTCCGCCTTCGGTCTTG-3′
Mouse *Myh3*-F	5′-ATGAGTAGCGACACCGAGATG-3′
Mouse *Myh3*-R	5′-ACAAAGCAGTAGGTTTTGGCAT-3′
Mouse *Ckm*-F	5′-AGACAAGCATAAGACCGACCT-3′
Mouse *Ckm*-R	5′-AGGCAGAGTGTAACCCTTGAT-3′

### Overexpression and RNAi

The overexpression and RNAi for human muscle stem/progenitor cells were performed by adenovirus infection. The cDNA of *ESR1* or *CREB* were cloned into the *Sal* I-*Eco*RV site of adenoviral vector pAdTrack-CMV (Addgene, cat#16405) for overexpression experiments. The adeno-backbone plasmid pAdTrack-U6 expressing shRNA (GCATTCTACAGGCCAAATTCA) was constructed and used for *ESR1* knockdown experiments. These vectors were first linearized by *Pme*I and then introduced into BJ5183-AD-1 cells. The recombinant adenoviral plasmids were transfected into 293 cells by Lipofectamine 2000 (Thermo Fisher Scientific, cat#11668019). The recombinant viruses were propagated in 293 cells and purified by CsCl gradient centrifugation for human muscle stem/progenitor cells infection. 1.2 × 10^6^ unit/μL virus was applied to 3 × 10^5^ human muscle stem/progenitor cells. These cells were subjected for various assays 48 h after infection.

### ELISA assay

ELISA assay to quantify ESR1 for human stem/progenitor cells was performed using Human Total ER alpha/NR3A1 DuoSet IC ELISA Kit (R&D, cat#DYC5715) following the manufacturer’s instructions. Total protein extracted from the isolated stem/progenitor cells was used.

### EdU Labeling

10 µM EdU (RiboBio, Cat#C10310-3) was applied to proliferating cells and labeled for 12 h. The cells were fixed by 4% PFA for 30 min and permeabilized by 0.5% Triton X-100 for 10 min at room temperature. Then the cells were stained with Apollo reaction cocktail (RiboBio, cat#C10310-3) and 1 μg/mL DAPI. Finally, samples were mounted with antifade mounting media for subsequent imaging. All images were analyzed and evaluated by investigators in a blinded manner.

### Luciferase assay

The potential promoter region of *MYH1* (-1958bp to transcription start site) or *MYH3* (-1536bp to transcription start site) was inserted into pGL3-basic vector (Promega). 293 T cells were transfected in triplicates by firefly luciferase reporter plasmid, renilla luciferase reporter plasmid and pCMV-*CREB* construct. Cells were harvested 48 h after transfection and lysed by Passive Lysis Buffer (Promega, cat#E1910). Luciferase activity was measured using Dual Luciferase Reporter Assay System in Glomax (Promega).

### Immunoblotting

Total proteins were extracted and resolved by SDS-PAGE, transferred to nitrocellulose membranes, blocked by 5% skim milk in 1× TBST buffer, and incubated with the primary antibody at 4 °C overnight. The following primary antibodies were used: anti-GAPDH (Cell Signaling Technology, cat#2118 L, 1:5000), anti-AKT (Cell Signaling Technology, cat#4685 s, 1:2000), anti-phospho-AKT (Cell Signaling Technology, cat#4060 s, 1:2000) anti-CREB (Proteintech, cat#12208-1AP, 1:1000), anti-phospho-CREB (Cell Signaling Technology, cat#9198 S, 1:1000), anti-estrogen receptor alpha (Abcam, cat#ab32063, 1:1000). HRP-conjugated secondary anti-rabbit IgG (Santa Cruz, cat#sc-2357, 1:10,000) was next incubated for 1 h at room temperature. Signals were visualized with ECL Reagent (ShareBio, cat#SBWB012) using GelDoc XR (Bio-Rad).

### In vivo muscle force analysis

The 1300 A 3-in-1 whole animal system (Aurora Scientific) was used for in vivo muscle force analysis. Mice were first anesthetized and kept warm by heat lamp. The maximum twitch and tetanic force were evaluated according to manufacturer’s instruction. Five repetitive tests were performed for each limb and DMA software (Aurora scientific) was used for results analysis.

### X-ray assessment

Anesthetized mice were subjected for X-ray spinal alignment evaluation. A standard imaging position was strictly performed for each mouse^56,57^. To avoid positional rotation during coronal Cobb angle measurement, mice were kept in prone position and fixed on a customized slant angled 30 degrees from the horizontal, with head, bilateral shoulders and bilateral lower extremities in neutral position. For sagittal kyphosis evaluation, mice were kept in a lateral decubitus position without extra traction. The X-rays of both coronal and sagittal images were obtained by Faxitron X-ray specimen radiography system (MX-20, USA) at the setting 10 s, 32 kV. Two board certified spine surgeons who were blind to sample group evaluated the coronal and sagittal angle independently. There was high interobserver reliability among them with intraclass correlation coefficient more than 0.95.

### Micro-CT assessment

Mice were sacrificed and the whole body was fixed in 4% PFA overnight. Then the mice were placed in an upright posture and fixed in scanner unit, with head, bilateral shoulders and bilateral lower extremities in neutral position. The spinal alignment was scanned by SkyScan 1272 (Bruker), and the 3D reconstruction of spine was then performed by 3D.SUITE (Bruker).

### Statistical analysis

All data were presented as mean ± SD. Each experiment was independently repeated for at least three times. Two-tailed Student’s *t*-test was performed for comparisons between groups using SPSS version 19.0 or GraphPad Prism 7. It was considered significant with *P* value less than 0.05. *P < 0.05; **P < 0.01; ***P < 0.001; ns, indicated no significant changes.

## Supplementary information

consents blank

IRB approval animal experiments

Supplemental Material

Signed consents

## Data Availability

The single-cell RNA-seq data have been deposited to Sequence Read Archive (SRA) database. The accession number is PRJNA722100. All other data are available in the main text or the supplementary materials.
