# Supplementary material for: The asymmetrical ESR1 signaling in muscle progenitor cells determines the progression of adolescent idiopathic scoliosis
Source: Cell Discov. 2023 Apr 25;9:44. doi: 10.1038/s41421-023-00531-5 (PMC10130095; doi:10.1038/s41421-023-00531-5)
Supplement: Supplementary file 2 — IRB approval animal experiments [file 41421_2023_531_MOESM2_ESM.pdf]

上海交通大学医学院附属新华医院科研项目知情同意书

研究者: **杨军林**

项目名称: **青少年特发性脊柱侧弯病人双侧椎旁肌病变研究**

基金支持: **国家自然科学基金 (No.82072519)**

尊敬的家长或法定监护人:

您的孩子将被邀请参加一项研究：**青少年特发性脊柱侧弯病人双侧椎旁肌病变研究**。您的孩子是否参加这项研究完全出于自愿。本知情同意书将向您提供关于该研究的重要信息，在决定是否参加研究之前，请您仔细阅读。如有任何问题或不明白的地方，请询问该研究项目的研究医生，研究团队将会回答您所提出的所有问题。如果同意参加，您需要在这份知情同意书上签字，并受到一份已签署的本文件副本。

本项研究的研究背景和研究目的是什么?

青少年特发性脊柱侧弯是一种在青春期女性中好发的儿骨科常见疾病，但病因未知。本次研究的目的是通过比较青少年特发性脊柱侧弯两侧椎旁肌病理改变，以先天性脊柱侧弯、脊柱肿瘤/创伤为对照，研究青少年特发性脊柱侧弯的病因机制。

研究执行机构和入组人数?

本项研究将在上海交通大学医学院附属新华医院脊柱中心实验室进行，预计招募符合标准的参与者 50 人。

如果参加研究，将会发生什么?

如果您和您的孩子决定参加本项研究，您孩子术中废弃的椎旁肌组织会被研究者收集并进行特发性脊柱侧弯病因学相关的进一步研究。

参加本研究有什么获益吗?

参加本研究可能不会给您和孩子带来短期内直接的获益。但您的参与可以帮助我们研究青少年特发性脊柱侧弯的病因，如果病因学研究有进展，那么很可能建立新的治疗方法，远期使得青少年特发性脊柱侧弯患者获益。

本项研究中可能存在的风险和不适有哪些？

本项研究收集的是术中废弃的肌肉组织，因此不会对参与者增加额外的风险和不适。

如何保护参与者的隐私和个人信息？

研究者建立保密措施和样本提供者信息安全制度，安全保存样本和数据，对在库所有样本和/或数据设置访问权限。当样本或制备的研究样本转移给其他研究者或机构时，对所有采集的样本采取匿名化管理(编码管理和删除全部身份识别信息)。研究所得数据可能公开发表或公布，但不会公布您与您孩子的姓名或可辨识身份的个人信息。

参与者的信息（生物学信息与个人隐私）会被用作本课题之外的研究吗？

参与者的生物学信息与个人隐私严格保密，不会被用作本课题之外的其他研究。

参加该项研究参与者是否获得报酬？

参与这项研究不会给予额外补助或者报酬。

样本提供者的自主决定权和撤销权

您有权拒绝签署本知情同意书，您不会因此受到歧视和差别对待，也不会影响您的正常诊疗。您同意提供后还可申请撤销，样本不再采集和利用、保存来源于您的生物样本，并在一段时间内保留销毁记录以备查询。

如果还有其他问题可以联系谁？

如果您对本研究或者参与者的权利存在疑问，您可以联系：杨军林教授，手机号码：19821843911，电子邮箱：yangjunlin@xinhuamed.com.cn.

上海交通大学医学院附属新华医院科研项目知情同意书

研究者: **杨军林**

项目名称: **青少年特发性脊柱侧弯病人双侧椎旁肌病变研究**

基金支持: **国家自然科学基金 (No.82072519)**

参与者知情同意声明

我已阅读并理解本知情同意书中的信息，并有机会提出问题，对所有问题的回答感到满意。我理解参加本项目完全是自愿的。我有权退出，任何医疗待遇与权益不会因此而受到影响。我会拿到一份已经签字的知情同意书副本。如果我有任何问题，我可以随时给研究医生打电话联系。

□ **我同意（允许我的孩子）参加这项研究**

□ **我同意研究样品继续保存**

□ **我同意研究样品转移外用**

参与者签名：______________ 父母/监护人签名: ______________ 日期:_________________

医院告知信息者声明

我已经准确地向样本提供者的父母/监护人解释了知情同意书的全部内容，回答了其所提出的所有问题。并提供一份签署过的知情同意书副本或复印件。

告知者签名: ___________________ 日期: _________________________

Consent Form for Participation in a Research Study

Xinhua Hospital Affiliated to Shanghai Jiaotong University School of Medicine

---

**Researcher(s):**	**Dr. Junlin Yang**
**Study Title:**	**Pathological changes of bilateral multifidus in adolescent idiopathic scoliosis**
**Funding:**	**The Natural Science Foundation of China (No.82072519)**

WHAT IS THIS FORM?

This form is called a Consent Form. It will give you information about the study so you can make an informed decision about participation in this research. If you decide to participate, you will be asked to sign this form and you will be given a copy for your records.

WHY ARE WE DOING IN THIS RESEARCH STUDY?

Adolescent Idiopathic Scoliosis (AIS) is a common pediatric skeletal disease highly occurred in females, while the pathogenesis of AIS has not been fully elucidated. We are conducting this research study to investigate the pathological changes of bilateral multifidus in AIS by comparing with patients diagnosed as congenital scoliosis (for control), spine trauma or tumor (for control).

WHERE WILL THIS RESEARCH STUDY TAKE PLACE AND HOW MANY PEOPLE WILL PARTICIPATE?

The research will be conducted in the Laboratory of Spine Center, Xinhua Hospital affiliated to Shanghai Jiaotong University, Shanghai, China. Approximately fifty participants are expected to be enrolled.

WHAT WILL I BE ASKED TO DO?

With your permission, the discarded paraspinal muscle from your child’s operation will be donated for further investigation of the etiology of AIS.

WILL BEING IN THIS RESEARCH STUDY HELP ME IN ANY WAY?

You may not be directly benefited from this research in the short term. However, the research results with your generous donation will help clarifying the etiology of AIS. If the etiology is further understood, it is possible that novel treatment can be established to benefit AIS patients.

WHAT ARE MY RISKS OF BEING IN THIS RESEARCH STUDY?

There is no additional risk of current research study since the harvested paraspinal muscle from your child's operation will be discarded routinely if you do not choose to donate it.

HOW WILL MY PERSONAL INFORMATION BE PROTECTED?

Your privacy and confidentiality are important to us. The researchers will keep all study records, including any codes to your data, in a secure location. Research records will be labeled with a code. At the conclusion of this study, the researchers may publish their findings. Information will be presented in summary format and you will not be identified in any publications or presentations.

WILL MY INFORMATION (BIOSPECIMENS OR PRIVATE INFORMATION) BE USED FOR OTHER RESEARCH IN THE FUTURE?

Your information or biospecimens will not be used or distributed for any other future research studies.

WILL I BE GIVEN ANY MONEY OR OTHER COMPENSATION FOR BEING IN THIS RESEARCH STUDY?

There is no money or other compensation for being in this research study.

WHAT HAPPENS IF I SAY YES, BUT I CHANGE MY MIND LATER?

You do not have to be in this study if you do not want to. If you agree to be in the study, but later change your mind, you may inform us at any time. There are no penalties or consequences of any kind if you decide that you do not want to participate.

WHO CAN I TALK TO IF I HAVE QUESTIONS?

If you have any questions concerning your rights as a research subject, you may contact Dr. Junlin Yang at 19821843911 or yangjunlin@xinhuamed.com.cn.

Consent Form for Participation in a Research Study

Xinhua Hospital Affiliated to Shanghai Jiaotong University School of Medicine

**Researcher(s):**	**Dr. Junlin Yang**
**Study Title:**	**Pathological changes of bilateral multifidus in adolescent idiopathic scoliosis**
**Funding:**	**The Natural Science Foundation of China (No.82072519)**

SUBJECT STATEMENT OF VOLUNTARY CONSENT

When signing this form, I agree to (allow my child) to enter this study voluntarily. I have had a chance to read this consent form, and it was explained to me in a language which I use. I have had the opportunity to ask questions and have received satisfactory answers. A copy of this signed Informed Consent Form has been given to me. I will contact the investigator If I have any questions.

□ *please tick* **I agree to (allow my child to) take part in this research**

□ *please tick* **I agree to samples being stored**

□ *please tick* **I agree to samples being exported**

Participant's Signature: ________ Parent/Guardian's Signature: _________ Date: ________________

INVESTIGATOR STATEMENT OF CONSENT

I certify that the above was explained verbally to the parent/guardian, and that she/he understands the nature and the purpose of the study and consents to the participation of the child in the study. She/he has been given opportunity to ask questions which have been answered satisfactorily.

Investigator's Signature: __________________ Date: ________________________
