# Supplementary material for: The asymmetrical ESR1 signaling in muscle progenitor cells determines the progression of adolescent idiopathic scoliosis
Source: Cell Discov. 2023 Apr 25;9:44. doi: 10.1038/s41421-023-00531-5 (PMC10130095; doi:10.1038/s41421-023-00531-5)
Supplement: Supplementary file 3 — Supplemental Material [file 41421_2023_531_MOESM3_ESM.pdf]

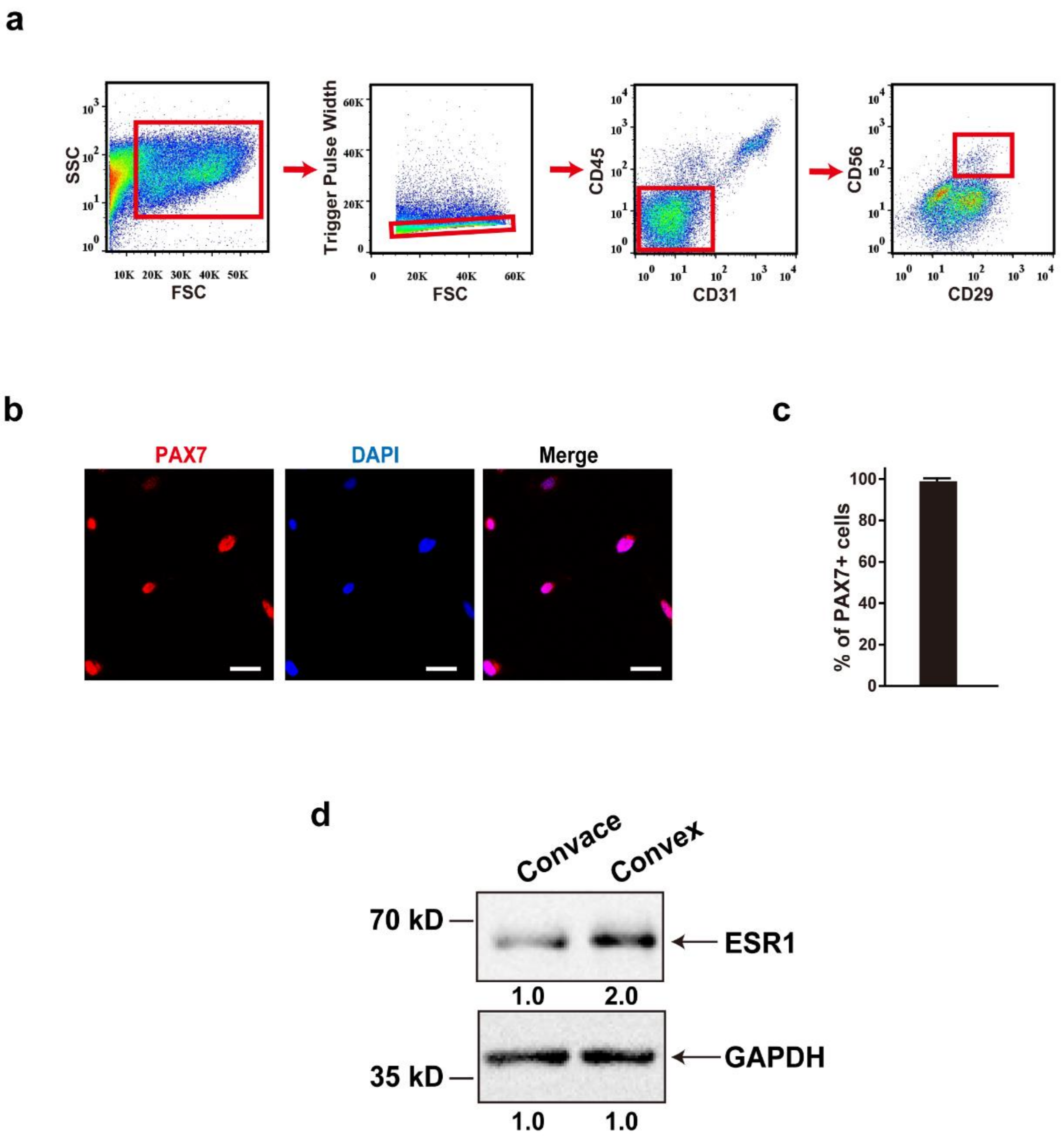

Supplementary Figure S1. (Related to Figure 1) Isolation of human muscle stem/progenitor cells.

a. Fluorescence activated cell sorting of human muscle stem/progenitor cells. After excluding debris and doublets, CD31- CD45- CD29+ CD56+ human muscle stem/progenitor cells were sorted.

b. Representative PAX7 immunofluorescent staining of human muscle stem/progenitor cells isolated from human para-spinal biopsies. Human muscle stem/progenitor cells were isolated by FACS sorting and were stained 6 hours after sorting. Red indicated PAX7; Blue indicated DAPI staining of nuclei; merge indicated the merged images of red and blue. Scale bars: 25µm.

c. Statistical analysis of the PAX7+ cells. Error bars indicated standard deviation (n=5).

d. Immunoblotting of ESR1 in muscle stem/progenitor cells isolated from para-spinal muscles for AIS patients. GAPDH served as internal control.

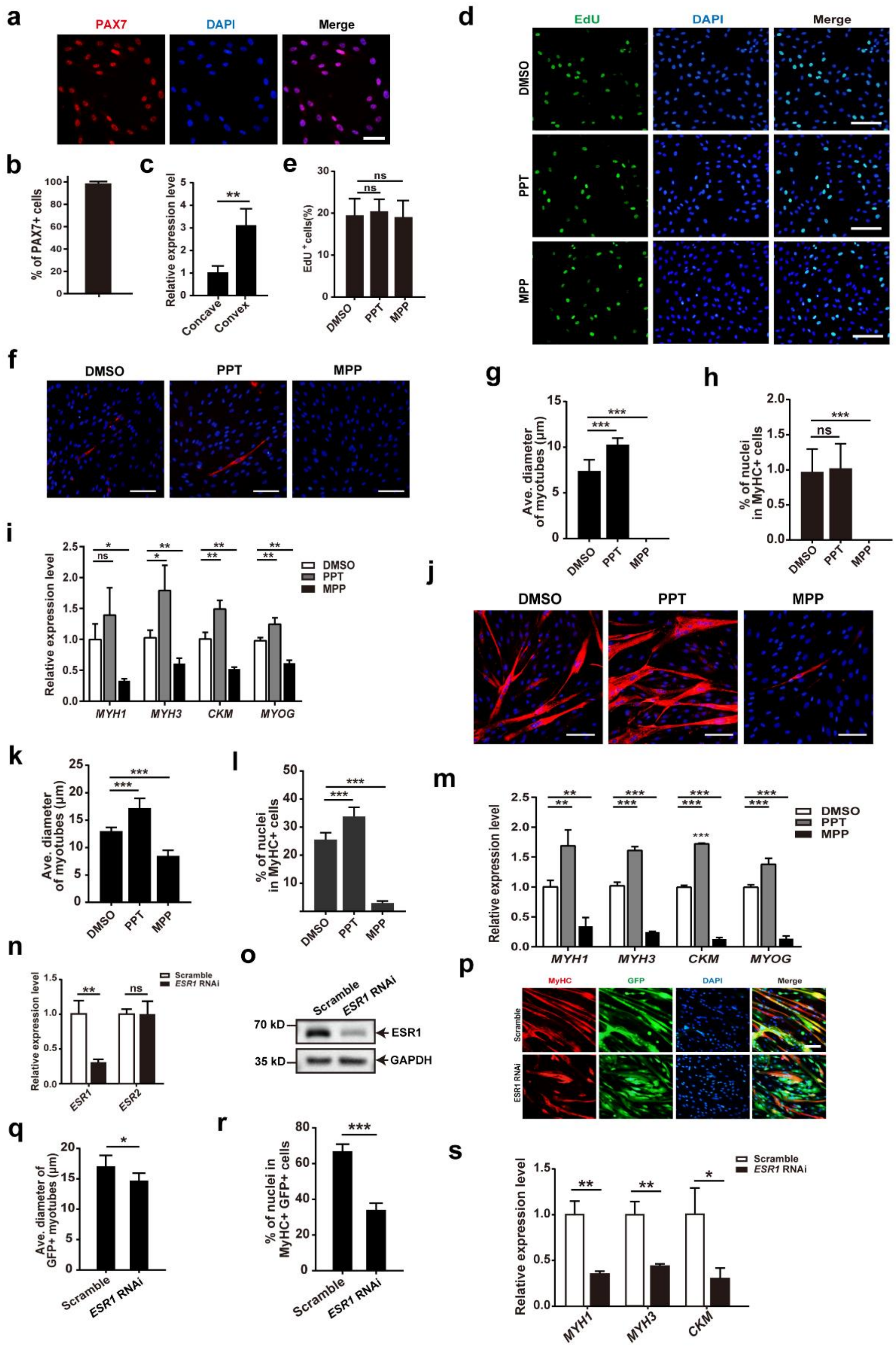

Supplementary Figure S2. (Related to Figure 2) ESR1 is required for human muscle stem/progenitor cell differentiation.

a. Representative PAX7 immunofluorescent staining of cultured human muscle stem/progenitor cells. Human muscle stem/progenitor cells were cultured for 7 days after isolation. Red indicated PAX7; blue indicated DAPI staining of nuclei; merge indicated

the merged images of red and blue. Scale bars: 50μm.

b. Statistical analysis of the PAX7+ cells. Error bars indicated standard deviation (n=5).
c. Expression level of ESR1 in human muscle stem/progenitor cells isolated from bilateral sides of the AIS patients. Error bars indicated standard deviation based (n=3). ** indicated $p<0.01$.
d. EdU pulse labeling of human muscle stem/progenitor cells treated by ESR1 antagonist or agonist. Human muscle stem/progenitor cells were treated by EdU for 12 hours and subjected for immunofluorescent staining. Green indicated EdU; blue indicated DAPI staining of nuclei; merge indicated the merged images of green and blue. Scale bars: 100μm
e. Statistical analysis of percentage of EdU positive cells during cell proliferation with different treatments. Error bars indicated standard deviation (n=5). ns indicated no significant changes.
f. Immunofluorescent staining of differentiating human myoblasts treated with DMSO, ESR1 agonist PPT, or ESR1 antagonist MPP for 1 day. Red indicated MyHC; Blue indicated DAPI staining of nuclei. The merged images were shown. Scale bars: 100μm.
g. Statistical analysis of average diameters of myotubes 1 day after differentiation. Error bars indicated standard deviation (n=5). *** indicated $p<0.001$.
h. Statistical analysis of differentiation efficiency day 1 after differentiation. Error bars indicated standard deviation (n=5). *** indicated $p<0.001$.
i. Expression levels of differentiation markers day after differentiation. Total RNA was extracted from differentiating cells treated by DMSO, ESR1 agonist PPT, or ESR1 antagonist MPP for 1 days followed by RT-qPCR analysis. Error bars indicated standard deviation (n=3). *** indicated $p<0.001$, ** indicated $p<0.01$.
j. Immunofluorescent staining of differentiating human myoblasts treated with DMSO, ESR1 agonist PPT, or ESR1 antagonist MPP for 3 days. Red indicated MyHC; Blue indicated DAPI staining of nuclei. The merged images were shown. Scale bars: 100μm.
k. Statistical analysis of average diameters of myotubes. Error bars indicated standard deviation (n=5). *** indicated $p<0.001$.
l. Statistical analysis of differentiation efficiency 3 days after induction. Error bars indicated standard deviation (n=5). *** indicated $p<0.001$, ns indicated $p>0.05$.
m. Expression levels of differentiation markers 3 days after induction. Total RNA was extracted from differentiating cells treated by DMSO, ESR1 agonist PPT, or ESR1 antagonist MPP for 3 days followed by RT-qPCR analysis. Error bars indicated standard deviation (n=3). ** indicated $p<0.01$, *indicated $p<0.05$, ns indicated $p>0.05$.
n. Expression levels of *ESR1* and *ESR2* after RNAi against *ESR1*. Human muscle stem/progenitor cells were infected by adenovirus encoding *GFP-ESR1-shRNA* for 2 days. Error bars indicated standard deviation (n=3). ** indicated $p<0.01$; ns indicated no significant changes.
o. Representative immunoblotting of ESR1 in *ESR1* RNAi human muscle stem/progenitor cells. Total proteins were extracted from human muscle stem/progenitor cells infected by adenovirus encoding *GFP-ESR1-shRNA* for 2 days and blotted for ESR1. GAPDH served as the internal control.
p. Representative MyHC staining of *ESR1* RNAi human muscle stem/progenitor cells.

Human muscle stem/progenitor cells were infected by adenovirus encoding *GFP-ESR1-shRNA*. Cells were induced to differentiate for 6 days after infection and subjected for immunofluorescent staining. Red indicated MyHC; green indicated GFP; blue indicated DAPI staining of nuclei; merge indicated the merged images of red, green and blue. Scale bars: 100μm.

q. Statistical analysis of the average diameter of myotubes infected by adenovirus encoding *GFP-ESR1-shRNA*. Error bars indicated standard deviation (n=5). * indicated $p<0.05$.

r. Statistical analysis of differentiation efficiency. Error bars indicated standard deviation (n=5). ** indicated $p<0.01$.

s. Expression levels of *MYH1*, *MYH3*, and *CKM* in differentiated *ESR1* RNAi cells. Human muscle stem/progenitor cells were infected by adenovirus encoding *GFP-ESR1-shRNA* and induced to differentiated for 6 days. The differentiated cells were harvested for RT-qPCR. Error bars indicated standard deviation (n=3). * indicated $p<0.05$; ** indicated $p<0.01$.

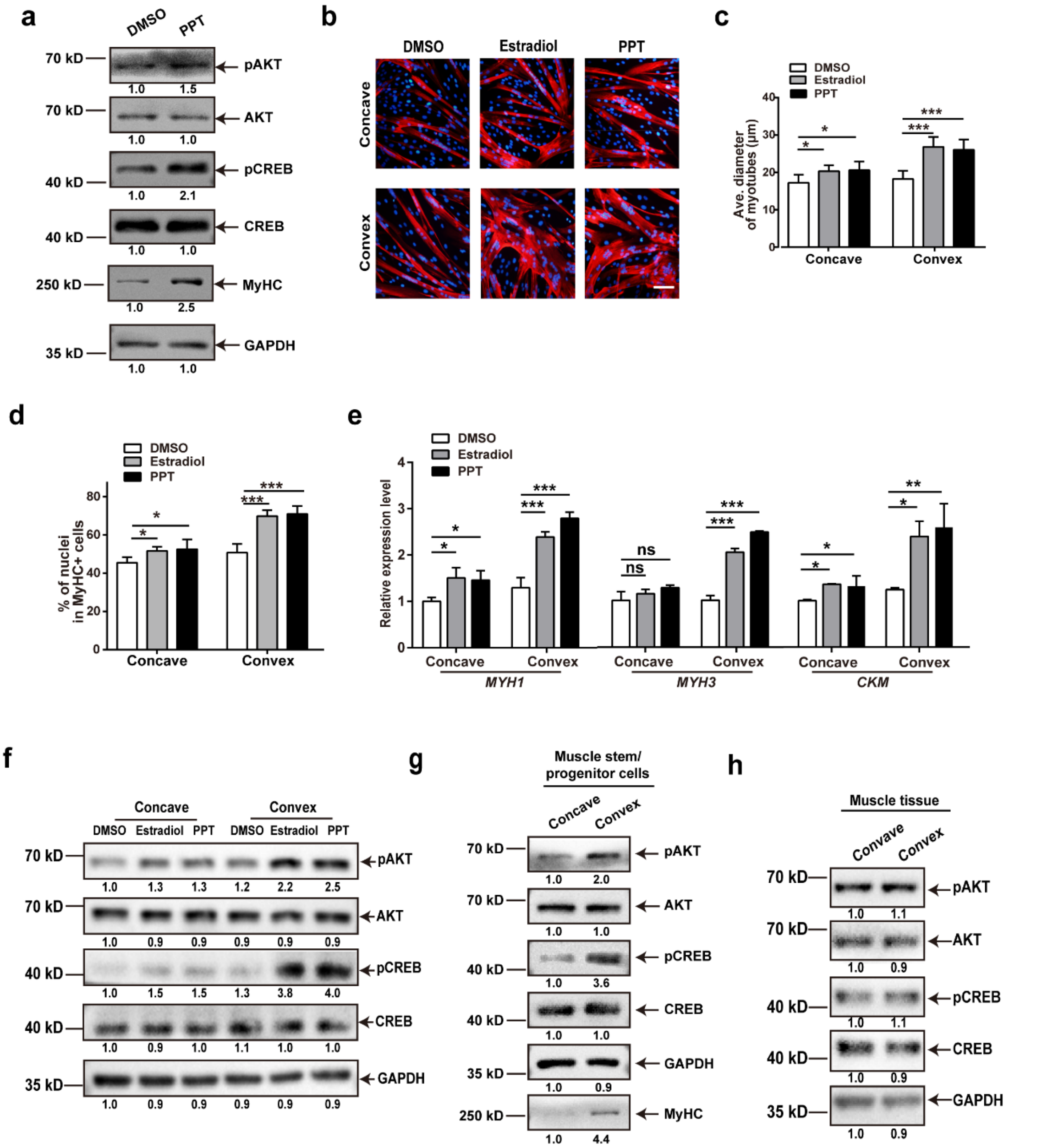

Supplementary Figure S3. (Related to Figure 3) ESR1 signaling is repressed in human muscle stem/progenitor cells at the concave side in AIS patients.

a. Protein levels of the key components in ESR1-AKT-CREB signaling pathway in human muscle stem/progenitor cells treat with ESR1 agonist PPT. Human muscle stem/progenitor cells treated by PPT or DMSO were harvested for immunoblotting analysis. GAPDH served as an internal control. The quantification of the band intensity was listed below each band.

b. Representative immunofluorescent staining of MyHC in differentiated human muscle stem/progenitor cells from AIS patients treated with ESR1 signaling activators (estradiol or PPT). Red indicated MyHC; green indicated GFP; blue indicated DAPI staining of nuclei; merge indicated the merged images of red, green and blue. Scale bars: 100μm.

c. Statistical analysis of the average diameters of the differentiated myotubes. Error bars indicated standard deviation (n=5). * indicated $p<0.05$; ** indicated $p<0.01$.

d. Statistical analysis of the differentiation efficiency. Error bars indicated standard deviation (n=5). * indicated $p<0.05$; ** indicated $p<0.01$,*** indicated $p<0.001$.
e. Expression levels of differentiation markers in human muscle stem/progenitor cells treated with estradiol or PPT upon differentiation. The differentiated cells were harvested after 6 day of differentiation and the expression levels of *MYH1*, *MYH3*, and *CKM* were analyzed by RT-qPCR. Error bars indicated standard deviation (n=3). * indicated $p<0.05$; ** indicated $p<0.01$; *** indicated $p<0.001$.
f. Protein levels of the key components in ESR1-AKT-CREB signaling in bilateral human muscle stem/progenitor cells isolated from AIS patients after ESR1 activator treatment. Upon initiation of differentiation, DMSO, estradiol, or ESR1 agonist PPT was added in culture, respectively. Cells were treated for 2 hours and harvested for immunoblotting analysis. The quantification of the band intensity was listed below each band.
g. Protein levels of the key components in ESR1-AKT-CREB signaling pathway in bilateral human muscle stem/progenitor cells isolated from AIS patients after differentiation induction. Cells were harvested for immunoblotting analysis. GAPDH served as an internal control. The quantification of the band intensity was listed below each band.
h. Protein levels of phosphorylated AKT and CREB in muscle tissues at concave and convex sides of AIS patients. GAPDH served as an internal control. The quantification of the band intensity was listed below each band.

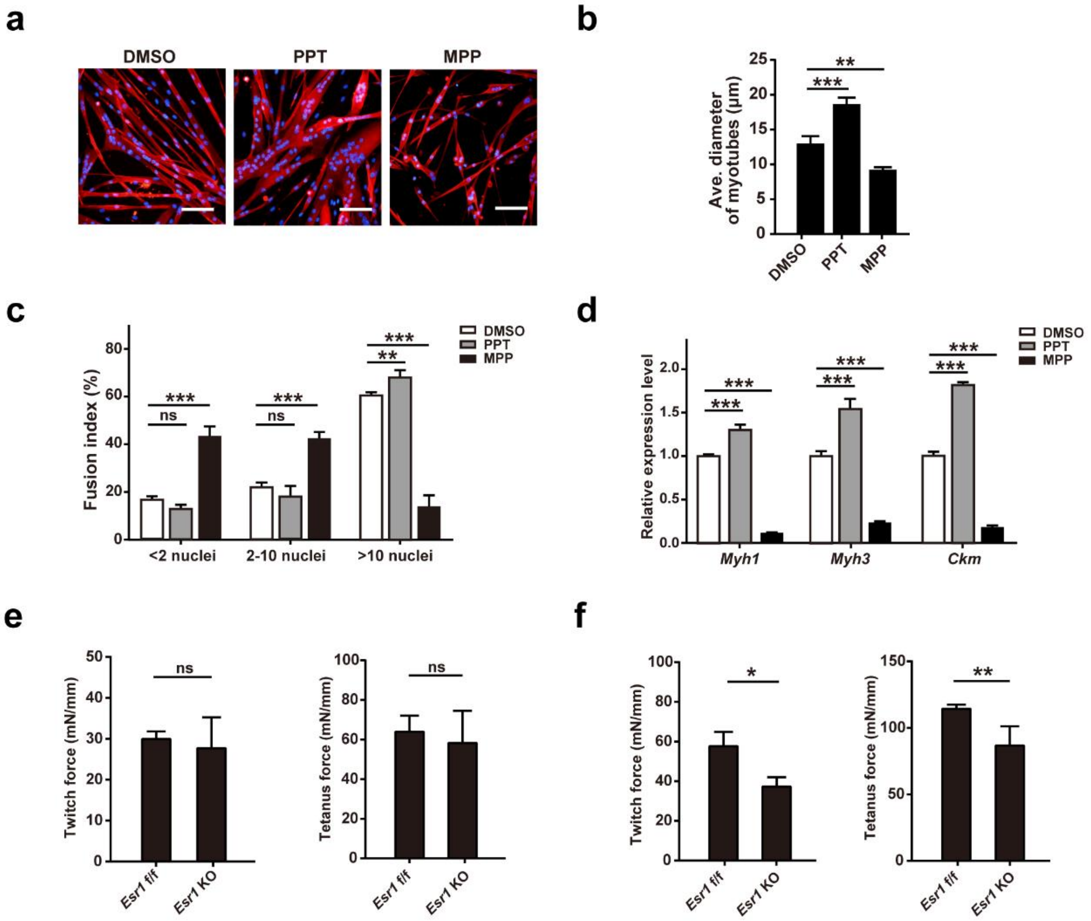

Supplementary Figure S4. (Related to Figure 4) MPP significantly inhibits differentiation in mouse muscle stem cells and *Esr1* KO in muscle stem cell impairs muscle strength.

a. Representative MyHC immunofluorescent staining in myotubes treated by ESR1 signaling agonist PPT and antagonist MPP, respectively. Mouse muscle stem cells were induced to differentiate for 3 days upon the treatment of PPT or MPP and analyzed by MyHC immunofluorescent staining. Red indicated MyHC; blue indicated DAPI staining of nuclei. The merged images of red and blue were shown. Scale bars: 100μm.

b. The statistical analysis of the average diameters of myotubes formed upon treatment with ESR1 signaling agonist or antagonist. Error bars indicated standard deviation (n=5). ** indicated $p<0.01$; *** indicated $p<0.001$.

c. The statistical analysis of the fusion index of myotubes after PPT or MPP treatment. Error bars indicated standard deviation (n=5). ** indicated $p<0.01$; *** indicated $p<0.001$; ns indicated no significant change.

d. The expression levels of differentiation markers after activation or repression of ESR1 signaling. Mouse muscle stem cells were differentiated for 3 days upon the treatment of PPT or MPP and the cells were harvested to analyze the expression levels of *Myh1*, *Myh3*, and *Ckm* by RT-qPCR. Error bars indicated standard deviation (n=3). * indicated $p<0.05$; ** indicated $p<0.01$; *** indicated $p<0.001$.

e. Statistical analysis of tibialis anterior muscle strength for *Esr1* f/f and *Esr1* KO mice at the age of 3 weeks old. Mice were treated with vehicle (*Esr1* f/f) or tamoxifen (*Esr1*

KO) every other day from 2 weeks old to 3 weeks old. Error bars indicated standard deviation (n=5). ns indicated no significant changes.

f. Statistical analysis of TA muscle strength for *Esr1* f/f and *Esr1* KO mice at the age of 8 weeks old. Mice were treated with vehicle (*Esr1* f/f) or tamoxifen (*Esr1* KO) every other day from 2 weeks old to 3 weeks old. Error bars indicated standard deviation (n=5). * indicated $p<0.05$, ** indicated $p<0.01$.

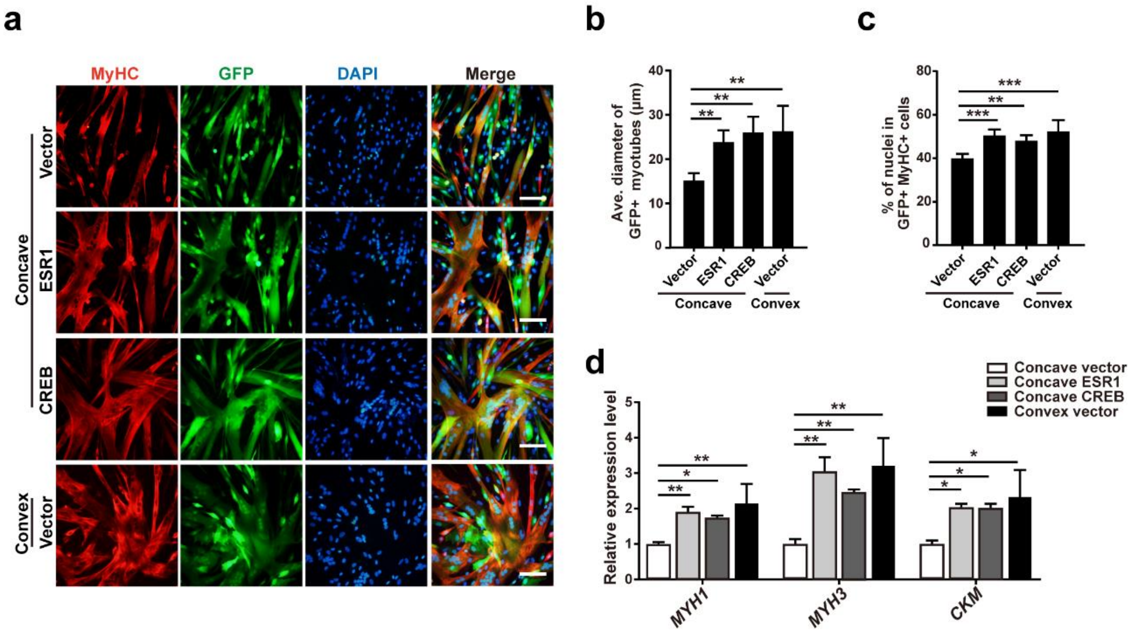

Supplementary Figure S5. (Related to Figure 5) Reactivation of ESR1-AKT-CREB-MYH signaling rescues the differentiation defects of concave muscle stem/progenitor cells.

a. Representative MyHC immunofluorescent staining of human muscle stem cells from para-spinal muscles in AIS with ESR1 or CREB overexpression. Human muscle stem cells were isolated from the concave side of the AIS patients and infected by adenovirus encoding *GFP-ESR1* or *GFP-CREB*. Three days after infection, the cells were induced to differentiate for 6 days and harvested for immunofluorescent staining. Bilateral human muscle stem/progenitor cells infected by empty adenovirus were served as control. Red indicated MyHC; green indicated GFP; blue indicated DAPI staining of nuclei; merge indicated the merged images of red, green, and blue. Scale bars: 100μm.

b. The statistical analysis of the average diameter of the myotubes overexpressing ESR1 or CREB. Error bars indicated standard deviation (n=5). ** indicated $p<0.01$; *** indicated $p<0.001$.

c. The statistical analysis of the differentiation efficiency. Error bars indicated standard deviation (n=5). * indicated $p<0.05$; ** indicated $p<0.01$; *** indicated $p<0.001$.

d. Expression levels of differentiation markers in human muscle stem/progenitor cells overexpressing ESR1 and CREB by RT-qPCR. Human muscle stem/progenitor cells isolated from the concave side were infected by adenovirus encoding *ESR1* or *CREB*. Three days after infection, the cells were induced to differentiate for 6 days. Bilateral human muscle stem/progenitor cells infected by empty adenovirus were served as control. Error bars indicated standard deviation (n=3). * indicated $p<0.05$; ** indicated $p<0.01$; *** indicated $p<0.001$.
