# Supplementary material for: The asymmetrical ESR1 signaling in muscle progenitor cells determines the progression of adolescent idiopathic scoliosis
Source: Cell Discov. 2023 Apr 25;9:44. doi: 10.1038/s41421-023-00531-5 (PMC10130095; doi:10.1038/s41421-023-00531-5)
Supplement: Supplementary file 4 — Signed consents [file 41421_2023_531_MOESM4_ESM.pdf]

上海交通大学医学院附属新华医院医学伦理委员会

Ethics Committee of Xin Hua Hospital Affiliated to Shanghai Jiao Tong University School of Medicine

Approval No. XHEC-D-2019-093

Ethical approval for "Pathological changes of bilateral multifidus in adolescent idiopathic scoliosis and its role in curve progression"

Dr. Junlin Yang et al. have submitted the research protocol for project "**Pathological changes of bilateral multifidus in adolescent idiopathic scoliosis and its role in curve progression**". The project aims to study the pathological changes of bilateral multifidus in adolescent idiopathic scoliosis and investigate the potential role of muscle imbalance in curve progression. In this protocol, patients with surgical treatment for adolescent idiopathic scoliosis, congenital scoliosis, spine trauma, or spine tumor at Spine Cencer, Xinhua Hospital affiliated to Shanghai Jiao Tong University School of Medicine will be enrolled in this study. The discarded multifidus muscle biospecimens during subperiosteal dissection and debridement will be harvested. All patients enrolled will be fully informed and sign consent forms. The biospecimens harvested in this protocol will only be used in this project. The study protocol has been fully reviewed by the ethics committee of Xinhua Hospital affiliated to Shanghai Jiao Tong University School of Medicine. This study has no risks for patients and the rights of the children enrolled in the project have been fully protected and respected. This study is fully in line with the Declaration of Helsinki. The ethic committee of Xinhua Hospital affiliated to Shanghai Jiao Tong University School of Medicine approves this study.

Ethics Committee of Xin Hua Hospital affiliated to Shanghai Jiao Tong University School of Medicine

October 25, 2019

地址：上海市杨浦区控江路 1665 号　邮编：200092　电话：021-25076143　传真：021-25078922

Address:No.1665 Kongjiang Road Shanghai, P.R China,Postcode:200092　Tel: +86-21-25076143　Fax: +86-21-25078922
